# Wheat chloroplast pangenome reveals frequent intramolecular recombination in the inverted repeat regions

**DOI:** 10.1186/s12870-025-07577-5

**Published:** 2025-11-27

**Authors:** Hua Zhang, Xuebing Qiu, Zhiliang Zhang, Jijin Zhang, You Qing, Yafei Guo, Xinyue Song, Chengzhi Liang, Yanling Sun, Yang Zhao, Changbin Yin, Jing Wang, Fei Lu, Mingsheng Chen

**Affiliations:** 1 Laboratory of Advanced Breeding Technologies, Institute of Genetics and Developmental Biology, Chinese Academy of Sciences, Beijing, China; 2 University of Chinese Academy of Sciences, Beijing, China; 3 CAS-JIC Centre of Excellence for Plant and Microbial Science (CEPAMS), Institute of Genetics and Developmental Biology, Chinese Academy of Sciences, Beijing, China

**Keywords:** Wheat, Chloroplast, Intra-recombination, Inheritance, Pan-genome

## Abstract

**Background:**

In eukaryotes, the mutation rate of the chloroplasts is lower than that of the nuclear genomes. Advances in Next-Generation Sequencing (NGS) and Third-Generation Sequencing (TGS) technologies, together with improvements in genome assembly algorithms, have substantially propelled research in chloroplast genomics. Although nearly 9,000 chloroplast genomes have been released, chloroplast population genetics for specific species remains unexplored. The chloroplast genome possesses a quadripartite structure consisting of a large single-copy (LSC) region, a small single-copy (SSC) region, and two inverted repeats (IRs). A longstanding question is why the maternally inherited chloroplast genome does not appear to suffer from the Muller’s ratchet effect. It has been hypothesized that intramolecular recombination within the chloroplast genome may counteract this effect; however, direct evidence for such recombination remains lacking.

**Results:**

We conducted chloroplast population data analysis in hexaploid wheat and its ancestral relatives. One the basis of the pan-genome constructed and phylogeny analysis of chloroplast genomes of all samples, we calculated the chloroplast diversity of hexaploid wheat (*π* = 0.0001) is the lowest among the three ploidy types tested. Additionally, we found that during the formation of hexaploid wheat, only the chloroplasts from tetraploid wheat were inherited. Moreover, *Aegilops tauschii* contributed solely as the paternal provider of nuclear genome material. In the chloroplast genome assembly, we assembled IRa (inverted repeat A) and IRb (inverted repeat B), revealing multiple insertion/deletion sequence differences between them. Importantly, we discovered that recombination occurs between the IR regions of the chloroplast genome. Frequent recombination results in two structural configurations existing in nearly equal proportions within a single sample. This phenomenon has led to an increase in the nucleotide diversity of the chloroplast IR region within the wheat population, which was originally low among species in *Poaceae*.

**Conclusion:**

This study demonstrates the feasibility of assembling chloroplast genomes using low-depth whole-genome sequencing (WGS) and confirms that the chloroplast genome of hexaploid wheat originates from tetraploid wheat rather than *Aegilops tauschii*. Furthermore, we provide evidence of frequent intra-molecular recombination in the chloroplast IR regions, leading to the coexistence of two equimolar inversion isomers. Despite strong purifying selection, recombination increases genetic diversity within the IR regions, facilitating adaptation and maintaining the functional stability of essential genes. Our findings highlight the role of recombination in balancing genetic stability and flexibility in chloroplast genome evolution, offering new insights into nuclear-cytoplasmic interactions and polyploid adaptation.

**Supplementary Information:**

The online version contains supplementary material available at 10.1186/s12870-025-07577-5.

## Background

During evolution, eukaryotes, represented by land plants, underwent an endosymbiotic event that resulted in the acquisition of chloroplast organelles. This event marked the beginning of the compartmentalization of genetic material between the nucleus and organelles. Fossil evidence of red algae dating back over 1.2 billion years [8] suggests that chloroplasts originated during this time, initially possessing several thousand genes similar to their contemporaries. The combined effects of genome reduction and gene transfer have led to the substantial shrinkage of the cyanobacterial endosymbiont genome, with thousands of genes either lost, deleted, or transferred to the host nuclear genome [5]. During this process, the majority of proteins responsible for chloroplast biogenesis and functional regulation are encoded by the nuclear genome, as demonstrated in a glaucocystophyte, a rhodophyte, a diatom, an euglenophyte, and various land plants [37, 43]. This was further validated in early experiments on protein synthesis and transport [13, 23].

To gain deeper insights into photosynthesis and chloroplast biogenesis, early studies sequenced the complete chloroplast genomes of several model plants [21, 36, 43, 48] revealing the typical quadripartite structure, which consisting of two copies of an inverted repeat (IR) separating two single-copy regions one is the large single-copy region (LSC), and other is the small single-copy region (SSC) [53]. The evolution, structure, and gene content of chloroplast genomes have been extensively reviewed [17, 58]. The smallest known chloroplast genome (11.35 kb) belongs to the African holoparasite *Pilostyles aethiopica* [6], while the largest chloroplast genomes in land plants (exceeding 200 kb) are found in the Geraniaceae family [18]. Compared to mitochondrial genomes, chloroplast genomes sizes are highly stable in angiosperms [45, 58].

Organelles are predominantly maternally inherited, thereby excluding them from the possibility of genetic recombination. According to Muller’s ratchet theory [39], this mode of uniparental inheritance renders organelle genomes evolutionary dead ends. In such systems,such as certain bacteria, viruses, and some unicellular organisms, deleterious mutations accumulate irreversibly over generations, progressively reducing the overall fitness of the population and potentially leading to extinction due to excessive mutational burden [3]. However, despite being asexual genetic systems, chloroplast genomes exhibit significantly lower mutation rates compared to nuclear genomes [59]. How plant organelle genomes mitigate mutation accumulation and thus slow the progression of Muller’s ratchet remains unclear.

The loss of genes and their transfer to the nuclear genome, a hallmark of chloroplast evolution, may have contributed to mitigating the effects of Muller’s ratchet [37, 55, 58]. To date, chloroplast genomes in *Poaceae* have remained within a size range of 120–160 kb and are among the least diverse. For instance, genes encoding photosystem I, the cytochrome b6/f protein complex, ATP synthase, ribosomal RNA, transfer RNA, RNA polymerase, and the majority of chloroplast biosynthetic genes are largely conserved. While genes such as *accD*, *ycf1*, and *ycf2* are conserved across nearly all land plants, they are uniquely absent in *Poaceae* [10, 29, 60].

Although the chloroplast genome is undergoing continuous reduction, the IR region in angiosperms is steadily expanding [57]. For instance, the IR expansion in *Nicotiana acuminata*, *Berberidaceae*, and *Lobelia thuliniana* has resulted in an extension of over 10 kb [16, 27]. An extreme case of sequence variation in the chloroplast genome is observed in some legumes, where one copy of the IR is lost. The loss of the IR has significant impacts on the stability of the chloroplast genome. For example, in *Medicago truncatula*, the loss of the IR has led to gene loss, genome rearrangements, and a mutation rate in this region that is 20 times higher than that of the single-copy regions. Similar hypervariable regions resulting from IR loss have been observed in other legume species. To investigate the functional role of the IR, Ralph et al. artificially removed one IR copy from the chloroplast genome of tobacco (*Nicotiana tabacum*) [28]. This manipulation increased the intracellular copy number of chloroplast genomes and an enhancement of the translation efficiency of chloroplast-encoded genes. Moreover, no significant phenotypic defects were observed in the plants. Although this compensatory dosage effect addresses the immediate phenotypic consequences in modern plants, it does not elucidate the role of IR in the evolutionary context of the chloroplast genome.

The IR region contributes to the stability of the chloroplast genome because, compared to single-copy regions, the inverted repeat regions diverge at a slower rate across different species [36]. For example, nucleotide diversity was estimated in 598 representative *Poaceae* chloroplast genomes, revealing that the IR region exhibited lower values compared to the LSC and SSC regions, indicating greater variation in the single-copy regions [25]. Whether this phenomenon is attributable to the duplicated structure of the IR region remains unclear. The capacity of the IR region to undergo recombination has long been a debated topic in chloroplast evolution. In asexual populations, such as bacteria, the lack of recombination can lead to the accumulation of deleterious mutations through Muller’s ratchet effect. If chloroplast genomes can recombine, it may partly explain their highly conserved nature. Evidence for IR recombination has been observed in algae from the genus *Osmunda*, where recombination in the IR region was demonstrated using restriction fragment length analysis [52]. This recombination resulted in the formation of two isomeric configurations, and their quantities were found to be equimolar. However, whether the IR region in plants can undergo intramolecular recombination remains an open question.

From the identification of the first chloroplast genome in higher plants, *Nicotiana tabacum* [53], to the current era driven by the reduced costs associated with high-throughput sequencing, nearly 11,000 chloroplast genomes have been released. This has spurred a rapid increase in the submission of chloroplast genomes to public sequence repositories. To date, this includes 6,000 plant species from the European alpine flora through the PhyloAlps project (https://www.france-genomique.org/spip/spip.php?article112), and 3,000 vascular plant species from the Arctic-boreal flora through the NorBol project (http://www.norbol.org/). Additionally, more than 2,000 chloroplast genome accession numbers for *Poaceae* are available in NCBI. Despite the vast number of chloroplast genomes released, analyses focused on chloroplast population data for specific species are lacking. Recent studies constructing chloroplast pangenomes have focused primarily on explaining interspecific differences in chloroplast genomes [34, 61, 64]. This study fills the research gap in understanding chloroplast inheritance bottlenecks, modes of inheritance, and genomic features within populations through population genetic analyses.

## Results

### Construction of the chloroplast pan-genome in wheat

**Fig.1:**
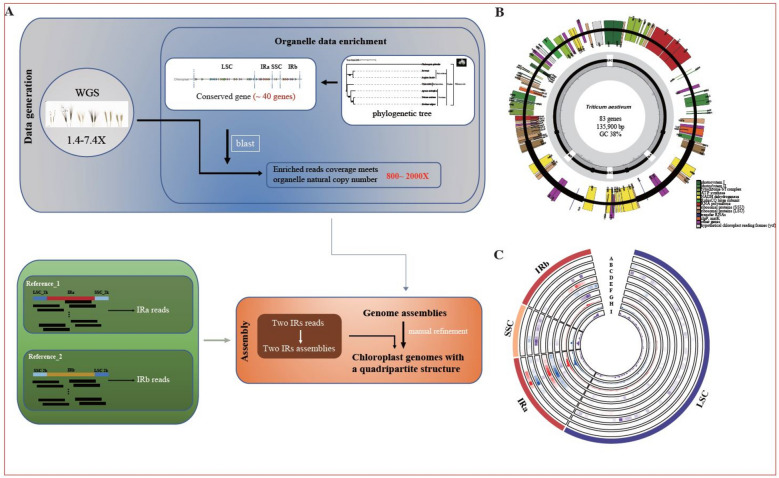
Construction of the chloroplast pan-genome in wheat. **A** Schematic diagram of chloroplast assembly process in wheat. We selected six *Poaceae* and one *Phalaenopsis aphrodite* as outgroups to analyze the conserved regions of the chloroplast genome (Supplementary Table 2). Approximately 40 conserved gene regions were evenly selected across the chloroplast genome to construct a reference set for the wheat chloroplast. Blast was used to enrich chloroplast sequences from whole-genome sequencing data. Additionally, we performed chloroplast sequencing data filtering and classified the raw data into IRa and IRb regions for separate assembly. **B** OGDRAW map of the Chinese Spring chloroplast genome. The gene location on the plus or minus strand is indicated by the outside or inside the circle, respectively. **C** Circos plot of wheat chloroplast pan-genome assembled from 61 accessions. a-c represent SNP density, d-f represent indel density, and g-i represent nucleotide diversity. Red indicates hexaploid wheat, blue indicates tetraploid wheat, and purple indicates *Aegilops tauschii*

**Fig.2:**
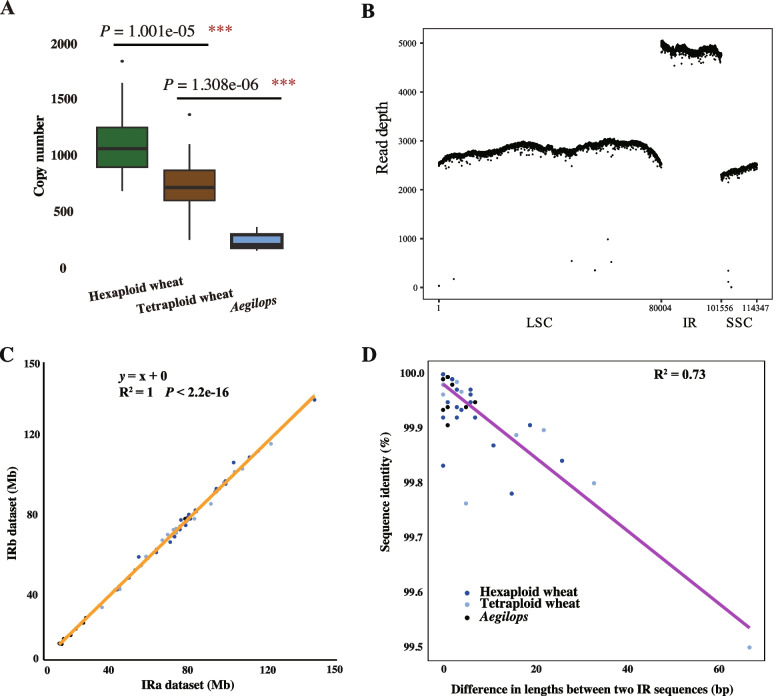
Evaluation of chloroplast genome assembly in wheat. **A** Calculation of chloroplast copy number in cells of wheat with different ploidy levels based on filtered data. Different ploidy levels of wheat exhibit distinct chloroplast copy numbers. Hexaploid wheat possesses the highest chloroplast copy number, whereas *Aegilops* shows the lowest. The differences in chloroplast copy number among wheat with different ploidy levels are highly significant. **B** Read depth of Chinese Spring chloroplast genome. To ensure the accuracy of reads mapping, we retained only one IR, and the reads were evenly distributed across the genome, with the IR exhibiting twice the coverage of the other SC regions. **C** Dot plot shows the dataset of IRa and IRb after classification. The correlation between the two datasets is 1, and the slope of the regression line is also 1, indicating that the sizes of the two datasets are identical. **D** The sequence differences between assembled IRa and IRb. As the length of sequence differences between the IRs increases, their identity decreases, indicating that the divergence between the IRs is primarily attributable to indels rather than SNPs

A total of 28 hexaploid wheat accessions, 24 tetraploid wheat accessions, and 9 *Aegilops tauschii* accessions were selected (Supplementary Table 1, 2). Due to the large nuclear genome of wheat, approximately 93% of the whole-genome sequencing data correspond to nuclear DNA [7], we established a streamlined workflow for the rapid isolation and enrichment of chloroplast sequencing data (Fig. 1A) (Supplementary Table 3). After filtering nuclear genome reads and excluding mitochondrial data, we obtained a set of 61 chloroplast genome assemblies (Supplementary Table 4). The filtered chloroplast sequencing reads were remapped to the wheat chloroplast reference genome, each achieving an average sequencing depth of over 1790 × (Supplementary Table 5). The coverage depth varied among wheat accessions of different ploidy levels, which was attributable to differences in the original sequencing depth of each sample (Extended Data Fig. 1A). Notably, over 99% of the chloroplast-enriched reads were properly mapped back to the chloroplast genome (Extended Data Fig. 1B), ensuring high accuracy and reliability for subsequent genome assembly. Here, we present the genome-wide read coverage of the chloroplast re-captured sequencing data for the *Triticum aestivum* cv. Chinese Spring (Fig. 2B). The coverage is uniform across the single-copy (LSC and SSC, SC) regions, whereas in the IR regions it is approximately twice that of the SC regions, consistent with the quadripartite structure of the chloroplast genome.

Previously, the released chloroplast genomes were believed to contain two IRs, IRa and IRb, which are identical in sequence but opposite in orientation [34, 61, 62, 64]. However, by separately classifying and assembling the IRa and IRb sequences from the raw sequencing data (Methods), we were able to reveal minor differences between the two regions. Further comparative analysis of the IR regions across our samples revealed several to dozens of sequence differences, which were scattered throughout the IR regions, mainly in the form of insertions/deletions (indels) (Fig. 2C, 2D). This finding is distinct from all previously released chloroplast genomes, which assumed that the IR regions were completely identical inverted repeats. Furthermore, our calculation of cpDNA copy numbers for each accession revealed results consistent with previous reports, showing a slight increase in cpDNA copy numbers per cell at higher ploidy levels [7]. Specifically, hexaploid wheat exhibited the highest cpDNA copy number per cell, *Aegilops tauschii* had the lowest, and tetraploid wheat displayed intermediate cpDNA copy numbers (Fig. 2A).

High-depth chloroplast sequencing data were utilized for chloroplast genome assembly. The assembly and annotation of 61 complete chloroplast genomes from wheat and its progenitor relatives were conducted (Supplementary Table 3, Methods). Figure 1B presents the visualization of the chloroplast genome of Chinese Spring. The total length of the chloroplast genome was 135,900 bp and included a pair of inverted repeats (IRs) regions (21,552 and 21,553 bp), one large single copy (LSC) region (80,004 bp), and one small single copy (SSC) region (12,791 bp). Interestingly, we identified a 1 bp difference between the IRa and IRb regions. Compared to the Chinese Spring reference genome, the sequence differences for each sample's chloroplast genome are shown in Extended Data Fig. 4A. These high-quality, complete assemblies provide a robust foundation for variant detection and genomic comparisons in constructing a chloroplast pangenome (Supplementary Table 6). Overall, we identified 1,298 high-quality variation sites in wheat chloroplast genome, including 649 single nucleotide polymorphisms (SNPs), 647 indels, and 2 structural variations (SVs) (Extended Data Fig. 4B, C). The proportions of SNP and indel variation in chloroplast genomes were similar, which contrasts with wheat nuclear genomes, where indels account for only 3.5%, with most variations being SNPs [2]. These variations were mainly concentrated in intergenic regions, while exonic regions exhibited the lowest number of variants. Notably, the proportion of indels in exonic regions was higher than that of SNPs. Further analysis of exonic indels revealed that 96 indels were located in exonic regions, with 8 distributed in the LSC region and 88 in the IR regions. Considering that genes in IR regions are present in two copies, this characteristic might mitigate the deleterious effects of indels in the IR region.

Analysis of variation density revealed its uneven distribution across the genome (Fig. 1C). Notably, the density of SNPs and indels in the IRs regions was higher than in the SC regions. Similarly, nucleotide diversity was greater in the IR regions compared to the SC regions. For instance, the nucleotide diversity of hexaploid wheat chloroplast genomes was highest in the IR regions, with IRa at 6.6 × 10⁻^4^ and IRb at 2.4 × 10⁻^4^, while the LSC and SSC regions exhibited lower diversity at 5.1 × 10⁻^5^ and 2.0 × 10⁻^5^, respectively. Interestingly, this pattern was consistent across accessions with different ploidy levels, indicating that the observed variation density differences primarily reflect regional genomic characteristics rather than ploidy-related effects. The elevated diversity in the IR regions suggests that these regions may be subject to different selective pressures compared to the SC regions within the chloroplast genome.

### Tetraploid wheat is the sole maternal progenitor in the speciation of hexaploid wheat

**Fig. 3:**
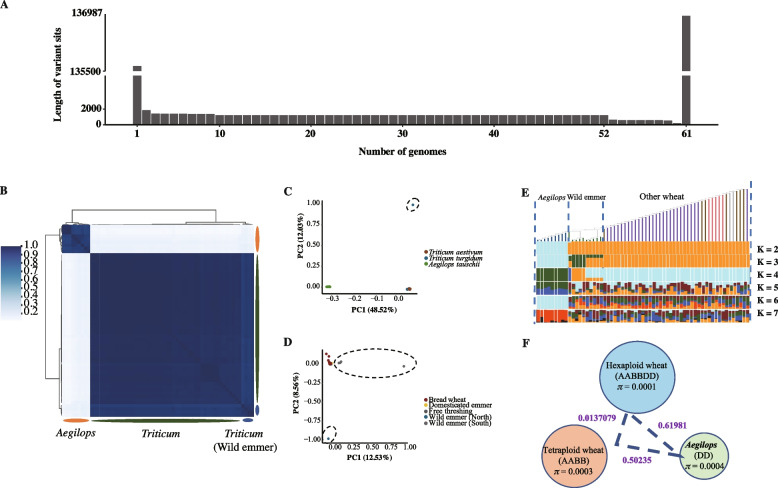
Pan-genome structure and phylogeny relationship of wheat chloroplasts. **A** Histogram of the sequence divergence information among samples. The first column represents the length of the Chinese Spring chloroplast genome, the last column represents the total non-redundant sequence length of all accessions, and columns 2–60 represent the maximum sequence divergence among any n-accession combinations. We randomly select n samples as a combination and integrate the unique sequence information from all the samples within the combination. Among different sample combinations, the longest unique sequence length was selected as the differential sequence length, which was then marked in the figure. **B** Heatmap map showing the clustering relationships among all accessions. The color gradually transitions from light blue to dark blue, indicating the similarity from low to high. *Aegilops*, *Triticum*, and *Triticum* (wild emmer) clustered into three distinct groups, respectively. **C** A PCA based on variants shows the clustering of accessions. The dashed line encloses a subset of tetraploid wheat accessions. **D** A PCA of tetraploid and hexaploid wheat accessions. The dashed line encloses all wild emmer accessions. **E** Ancestry coefficient analysis of the bread wheat. K varied from 2 to 7. Detailed information for the accessions is shown at the top. **F** Nucleotide diversity and population differentiation (*F*_ST_) in wheat chloroplast. The values in the circle represent nucleotide diversity, and numbers next to dashed lines represent *F*_ST_

Analysis of the pan-genome constructed from wheat chloroplast samples revealed that all chloroplast genomes have an identical number of genes, 83. Due to the much lower mutation rate of chloroplast genomes compared to nuclear genomes, the nucleotide diversity of wheat chloroplast genomes was also significantly lower than that of nuclear genomes. Unlike nuclear pan-genomes, which are presented based on different gene families, our chloroplast pan-genome provides a more detailed depiction of chloroplast information, enabling comprehensive sequence variation analysis across different samples at the whole-genome level (Fig. 3A). We established various sample permutations by randomly selecting samples and, within the limited set of permutations, assessed the total length of unique sequences in each combination that were not shared with other samples. We then compared and selected the maximum length of unique sequences across different combinations as the length of unique sequences for that sample. Based on the number of samples selected from different ploidy levels, we found that sequence differences between combinations of samples were substantially reduced when the sample size reached 52 (28 hexaploid wheat accessions plus 24 tetraploid wheat accessions) or more. This indicates that the chloroplast genome of *Aegilops tauschii* is more divergent from that of other wheat samples. Considering that *Aegilops tauschii* and tetraploid wheat are the ancestral species of hexaploid wheat, and given the maternal inheritance of chloroplast genomes, it is more likely that the chloroplast genome in hexaploid wheat was inherited from tetraploid wheat.

To investigate the origin of chloroplasts in hexaploid wheat, we conducted a clustering analysis. Consistent with the sequence variation results, *Aegilops* groups formed distinct clusters. A more detailed observation revealed that wild emmer (*Triticum dicoccoides*) also clustered independently (Fig. 3B), which aligns with the principal component analysis (PCA) results (Fig. 3C, D). Therefore, we conclude that wild emmer did not contribute to the speciation process of hexaploid wheat. Moreover, our results indicate that hexaploid wheat retains more tetraploid wheat chloroplast genome information, supporting the hypothesis that tetraploid wheat served as the maternal parent in its hybridization with *Aegilops tauschii*. To explore this phenomenon further, we analyzed the chloroplast population structure of hexaploid wheat. ADMIXTURE analysis showed that the deepest split (*K* = 2) occurred between two groups: one predominantly distributed in *Aegilops* and the other consisting of hexaploid and tetraploid wheat. From *K* = 3 to 4, all hexaploid wheat and tetraploid wheat (excluding wild emmer) exhibited mixed population structures, making finer population differentiation infeasible (Fig. 3E). We further calculated the genetic divergence (*F*_ST_) between *Aegilops* and different wheat groups. The *F*_ST_ value between the *Aegilops* chloroplast population and tetraploid wheat or hexaploid wheat populations were 0.5 and 0.62, respectively, while the *F*_ST_ between tetraploid and hexaploid wheat chloroplast populations was 0.01. More importantly, nucleotide diversity (*π*) estimates revealed that hexaploid wheat had the lowest diversity among the three groups (*π* = 0.0001). Based on these findings, we conclude that tetraploid wheat (excluding wild emmer) was the sole maternal contributor to the formation of hexaploid wheat.

This study investigated chloroplast diversity in wheat and its ancestral and related species, revealing that chloroplast diversity remains at approximately *π* = 0.0001, which is approximately one-tenth of the nuclear genome diversity (*π*_A_ = 0.0017, *π*_B_ = 0.0025, *π*_D_ = 0.0002) [65]. The D genome, due to its lack of introgression, exhibits a diversity level comparable to that of the chloroplast genome [65]. This low diversity is consistent with previous reports indicating that the D genome in bread wheat harbors substantially less variation than the A and B genomes. The latter likely reflects the fact that introgression from wild emmer (*Triticum turgidum* ssp. *dicoccoides*, AABB) has contributed to the genetic diversity of the A and B genomes [11, 22], whereas the D genome has experienced substantially less gene flow. Notably, we found that the chloroplast diversity of hexaploid wheat is not higher than that of its ancestral species (Fig. 3F). This is because the chloroplast genome of hexaploid wheat was solely inherited from tetraploid wheat, with no evidence of chloroplast genetic material from *Aegilops tauschii*.

### Frequent intramolecular recombination in the IR regions

**Fig. 4:**
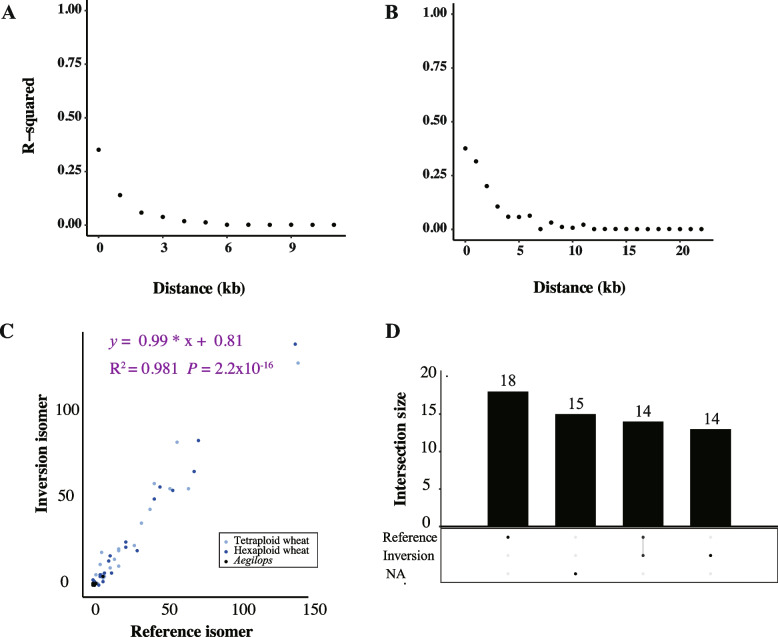
Intramolecular recombination between segments of the inverted repeat. **A** Linkage disequilibrium across the chloroplast IRa. **B** Linkage disequilibrium across the chloroplast IRb. *p* < 0.05 for all the measures. **C** The read counts of the supporting two isomers are generated by recombination of all accessions. Each point represents the depth of the isomer (reference isomer and inverted isomer) counts. **D** The UpSet plot displays the contig counts across all accessions for the chloroplast genome isomers. Fifteen contigs could not span the entire IR and could not be determined

The chloroplast genome structure of algae and land plants is highly conserved, with one notable exception of having deleted one-half of this inverted repeat in legumes [20, 30, 44, 56]. Apart from this, chloroplast genomes typically contain two IR sequences. Due to differences in inheritance patterns compared to the nuclear genome, chloroplast genomes are generally believed to lack recombination. The quadripartite structure of the chloroplast genome has long fueled debate over whether IR regions undergo recombination, making it a key focus of chloroplast evolutionary studies [46, 52, 63]. In this study, we conducted pairwise linkage disequilibrium (LD) analysis of the hexaploid wheat chloroplast genome based on its genomic regions (Supplementary Table 9). We detected signals of LD decay within the IR regions of the wheat chloroplast genome (Fig. 4A, B). In contrast, such recombination signals were absent in the whole chloroplast genome and other genomic regions (Extended Data Fig. 5). This provides evidence supporting the occurrence of intramolecular recombination in the IR regions of chloroplast genomes. Even though the SC region exhibits a trend of LD decay, this may be attributed to intramolecular recombination occurring between short repetitive sequences within the SC region [42]. A comparison between the SC and the two IR regions reveals that the starting point of r^2^ in the SC region is higher than that in the IR regions (r^2^ = 1). This is due to the overall lack of repetitive structures in the SC region, which suppresses recombination and maintains a high degree of LD. However, as the physical distance increases, the correlation between two mutations does not exhibit a gradual decline. In contrast, the linkage strength in the IR regions is lower at the degree of LD (r^2^ = 0.5). These findings collectively suggest that recombination has occurred in the chloroplast IR regions.

**Fig. 5:**
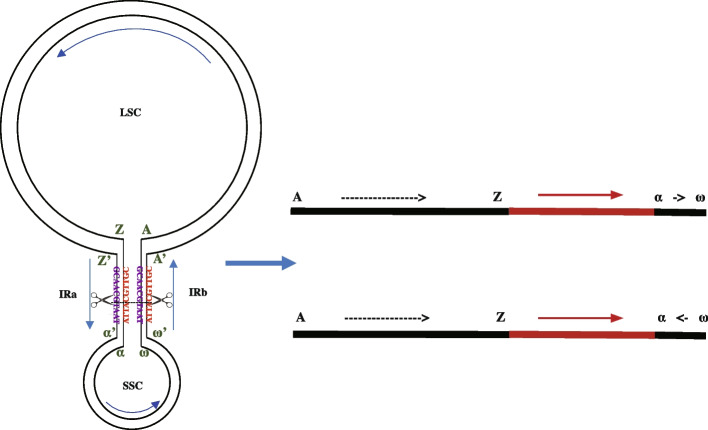
Two isomers formed by intramolecular recombination in chloroplast genome. Based on the coordinates of the chloroplast linear reference genome, the LSC sequence is labeled in the order of letters A-Z, with the outer ring labeled A’-Z’. The SSC is labeled in lowercase Greek letters α-ω, with the outer ring labeled α’-ω’. The direction of the arrows for both IRa and IRb indicates the sequence orientation of the inner circle

To further investigate the phenomenon and frequency of recombination in the IR regions of the chloroplast genome, we constructed two potential chloroplast genome inversion isomers that could exist following recombination events in the IR regions (Fig. 5). The two inversion isomers primarily differ in the orientation of the SC regions, as the sequences within the IR regions are nearly identical. While recombination does not cause significant changes in sequence composition, it alters the connection order between the IR and SC regions. By detecting the sequences of LSC and SSC adjacent to an IR region in the raw data, we determined the chloroplast genome isomer of the corresponding sequence. In all the samples, we observed the coexistence of two inversion isomers within a single sample (Fig. 4C), with a calculated ratio of 1:1. This indicates frequent intramolecular recombination in the IR regions, resulting in a uniform mixture of the two inversion isomers. To ensure accuracy, we applied stringent criteria requiring complete IR sequences and at least 2 kb of single-copy sequences on both flanking sides. Consequently, not all sequencing data from the samples could detect two inversion isomers. Similarly, we analyzed the contig sequences from the original assemblies based on the two inversion isomers (Fig. 4D). Among the 61 samples, contigs from 46 samples contained complete configuration sequences, whereas contigs from the remaining 15 samples were too short to determine any specific inversion isomer. Of the 46 samples with complete sequences, 18 supported only one isomer type (reference isomer), 14 supported only the other inversion isomer type (inversion isomer), and the remaining 14 supported both inversion isomers. No consistent correspondence was observed between the assembled contigs and the inversion isomers they supported, suggesting that the assembly process was random with respect to inversion type. We observed that sequence alignment orientations between the single-copy regions were not always consistent, providing further support for this conclusion. (Extended Data Fig. 6).

**Fig. 6:**
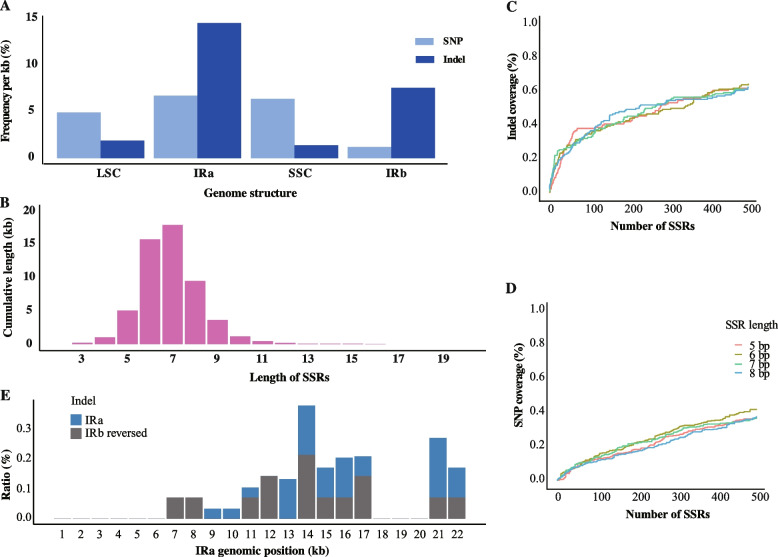
Distribution of mutation types mediated by intramolecular recombination. **A** The frequency of SNPs and indels in the four chloroplast genome structures. Light blue represents the SNP variations, and dark blue represents the indel variations. **B** Short, direct-repeat sequences in LSC. K-mers ranging from 3 to 30 bp in sliding windows were analyzed, and those between 2–20 bp were selected to illustrate the distribution of short, direct-repeat sequences in the LSC. **C** Proportion of indels explained by short direct-repeat sequences in the LSC. **D** Proportion of SNPs explained by short direct-repeat sequences in the LSC. The length of short direct-repeat sequences varied from 5 to 8 bp. **E** Position distribution of indel in IRa and IRb reversed sequence. Two IRs are inverted repeat sequences. Blue represents the position of IRa in the reference genome, while gray indicates the reversed sequencing of IRb to maintain sequence consistency with IRa

### Frequent recombination in the IR induces more indels

Based on our detection of recombination signals in the IR regions of the wheat chloroplast genome, combined with the observation that every individual sample contains two isomers generated by recombination, we prove that recombination indeed occurs in the IR regions of the chloroplast genome. Building on this finding, we further explored sequence variations in the chloroplast genome caused by recombination.

**Fig. 7:**
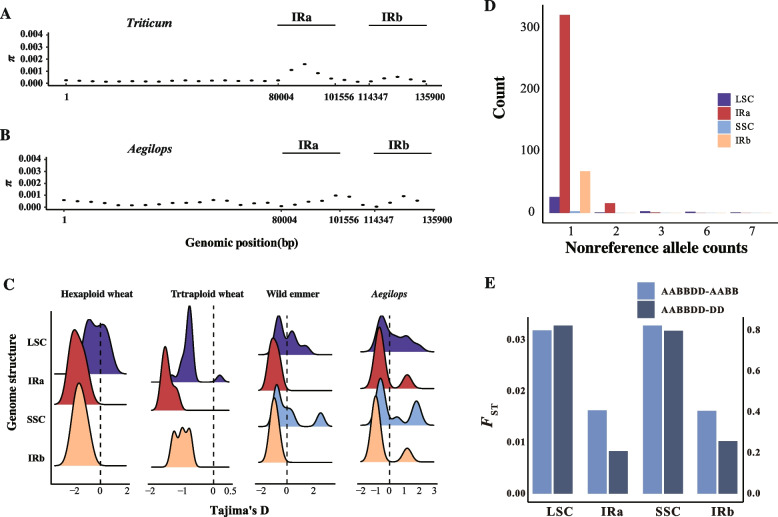
Selective pressure on different chloroplast genome structures. **A** Nucleotide diversity of *Triticum* chloroplasts across four genome structures. **B** Nucleotide diversity of *Aegilops* chloroplasts across four genome structures. **C** Distribution of Tajima’s D statistic for different chloroplast genome structures in *Triticum* and *Aegilops*. *Triticum* is divided into two populations, hexaploid wheat and tetraploid wheat. Additionally, wild emmer is recognized as an independent population separate from tetraploid wheat. **D** Distribution of allelic frequencies of four chloroplast genome structures in hexaploid wheat (*N* = 28). (E) *F*_ST_ values of the subpopulations in four chloroplast genome structures. Light blue corresponds to the values on the left y-axis, whereas dark blue corresponds to the values on the right y-axis

Although the number of SNPs and indels detected in the wheat chloroplast genome is comparable, their density distributions differ across the genome structure. For instance, indels are primarily concentrated in the IR regions (Fig. 1C), whereas SNPs exhibit a similar distribution between the IR and SC regions. We observed that rare variants are primarily enriched in the IR regions of the wheat chloroplast genome (Fig. 7D). A more detailed analysis of the types of rare variants revealed that they are predominantly indels (Extended Data Fig. 7). Indels account for half of the total variation in the wheat chloroplast population, but among rare variants, their number is three times that of SNPs. The rare variants enriched in the IR regions are mainly indels. This is likely because frequent recombination in the IR regions generates recombination footprints at breakpoints, typically 1–2 bp indels. This phenomenon has been widely reported in CRISPR experiments whether in plants or animals [4, 9, 47]. It aligns with our observation of a higher density of indels in the IR regions compared to SC regions (Fig. 6A). Although indels and SNPs formation mechanisms and their deleterious effects on genes differ, the IR regions consist of two highly repetitive segments in the chloroplast genome. In theory, every site in these regions could undergo intramolecular recombination, making it challenging to determine whether the repair of recombination breakpoints directly causes the formation of indels. We also observed a certain proportion of indels in the LSC region. Although the density of indels in the LSC region is lower than that of SNPs, the proportion of indels in the LSC region is higher than that in the nuclear genome (accounting for 3.5%). Considering the presence of numerous short repetitive sequences in the LSC region, intramolecular recombination between these repeats could occur [24, 42]. However, these results were derived from early comparisons between chloroplast genomes of different species and have not been correspondingly validated within populations. Therefore, investigating whether the formation mechanism of indels in the LSC region is related to intramolecular recombination may also provide insights into the occurrence of indels in the IR region.

We analyzed the short repeat sequences in the LSC region of the wheat chloroplast genome and found that most of them ranged from 5 to 8 bp in length (Fig. 6B). To avoid including overly simple repeats, we selected short repeats of 5–8 bp to examine the types of variant sites within this set of repetitive sequences. The results showed that short repetitive sequences in the LSC region accounted for 63% of the indels (Fig. 6C), whereas the proportion explained by SNPs was only 37.6% (Fig. 6D). The indels explained by short repetitive sequences in the LSC region were 1.6 times higher than SNPs. Therefore, we propose that the enrichment of indels in short repeat sequences may be attributed to intramolecular recombination events between these repeats.

With the higher explanatory power of short repetitive sequences in the LSC region for indels compared to SNPs, we hypothesize that indels in the LSC region may be introduced during intramolecular recombination. Based on this, indels are likely to cluster and exhibit consistent patterns at corresponding positions between IRa and IRb since they experience frequent recombination. To test this hypothesis, we analyzed the density distribution of indels in the IR region (Fig. 6E). The results show that the density distribution of indels in the IR region exhibits consistency at corresponding positions between IRa and IRb. These high-density regions of indels are likely hotspots of frequent recombination in the chloroplast IR region. In contrast, the distribution of SNPs in the IR region (Extended Data Fig. 8) does not exhibit such consistency between IRa and IRb. This discrepancy can be attributed to the different mechanisms by which indels and SNPs are generated, with indels being more likely introduced during intramolecular recombination events in the chloroplast genome.

### IR recombination enhances regional diversity within wheat populations

Previous studies have experimentally demonstrated that the chloroplast IR region in algae can undergo recombination. Our results provide direct evidence that frequent intramolecular recombination also occurs in the chloroplast IR region of land plants. The high density of indels in the IR region is linked to the recombination events occurring in this region. In addition, the ability of the overall IR structure to undergo recombination may result in evolutionary characteristics of the chloroplast genome that differ from those of the SC region. Notably, recombination increases the diversity of the IR region within populations (Fig. 7A, B), which contrasts with the greater conservation of the chloroplast IR region across different species [25]. Within populations, the chloroplast IR region exhibits higher diversity than the SC region due to recombination events that preserve more allele combinations within the IRs. Conversely, interspecies diversity in the chloroplast IR region is lower than in the SC region because recombination in the IRs maintains sequence consistency, while the SC region lacking recombination, exhibits greater sequence divergence among species.

To explore the differing evolutionary patterns of the IR region at the intra- and inter-species levels, we calculated Tajima's D for each structural component of the chloroplast genome. We divided the samples into four subgroups based on ploidy and the genetic distance of the chloroplast genome. Comparative analysis of the quadripartite structure revealed that Tajima's D values were all lower in the IR region than in the single-copy regions (Fig. 7C). When combined with the observation that the IR region exhibits increased diversity within the wheat chloroplast population, this suggests that the IR region may be subject to positive selection. However, we also observed a high proportion of rare variants in the wheat chloroplast genome, predominantly concentrated in the IR region (Fig. 7D). This indicates that the IR region is subject to stronger purifying selection than the SC regions. The more pronounced reduction in diversity in the IR region across species further supports the likelihood of purifying selection in this region.

To explain the higher diversity of the IR region within the wheat chloroplast population, we propose three possible causes. First, we assembled the IRa and IRb regions separately, resulting in a more comprehensive inclusion of sequence information from the IR region (Fig. 2C, 2D). Second, frequent intramolecular recombination within the chloroplast IR region may retain a greater variety of IRa-IRb site combinations, which subsequently expand within populations (Fig. 4, 5). Lastly, the genetic bottleneck associated with chloroplast inheritance may magnify the influence of genetic drift on the variation arising from frequent recombination in the IR region during *Poaceae* scale evolution. These factors likely contribute to the elevated diversity of the IR region at the population level. Further *F*_ST_ calculations for each chloroplast genome structure revealed a significant reduction in *F*_ST_ values within the IR region (Fig. 7E). This pattern was observed not only between hexaploid and tetraploid wheat populations but also between hexaploid wheat and *Aegilops tauschii* chloroplast populations. Although purifying selection acts on the chloroplast genome as a whole (Extended Data Fig. 9), the findings underscore the stronger purifying selection exerted on the IR region. In summary, while recombination in the IR region can enhance short-term diversity compared to the SC regions, this effect only occurs within populations. Purifying selection gradually diminishes this effect, as sublineages founded by different mutant individuals evolve independently. Considering that the frequency of indels introduced by recombination in the IR region is higher in gene regions than in the SC region, deleterious mutations are subject to natural selection, which prevents them from reaching frequencies much higher than a critical threshold. The frequency of a mutant lineage is influenced primarily by genetic drift [15]. Strong purifying selection over longer evolutionary timescales reduces diversity in the IR region among species. Our evidence consistently highlights the IR region as the most conserved part of the chloroplast genome (Extended Data Fig. 10). In *Poaceae*, chloroplast genomes maintain a GC content below 40%; for example, the Chinese Spring chloroplast genome has a GC content of 38%. However, certain segments of the IR region exhibit GC content exceeding 50%, which is substantially higher than other structural components of the chloroplast genome. Compared to nuclear genomes, the chloroplast genome is increasingly recognized for its greater conservation. Nonetheless, our findings indicate that the IR region is even more conserved, potentially linked to the intramolecular recombination events occurring within this segment.

## Discussions

### Chloroplast genome assembly from low-depth whole-genome sequencing

Genomic assembly typically requires high sequencing depth, however, for large genomes like that of wheat, assembling the chloroplast genome without high-depth whole-genome resequencing. Due to the small size of the chloroplast genome and its high copy number in leaf cells, complete assembly of the chloroplast genome can be achieved. In this study, low-depth whole-genome sequencing (approximately 1–3 ×) was performed on 61 wheat samples and their closely related ancestral species, utilizing the advantages of third-generation long reads to achieve rapid assembly of the chloroplast genome. We further evaluated the feasibility of assembling chloroplast genomes using low-depth whole-genome sequencing (WGS) data. Specifically, we tested the relationship between total sequencing depth and the corresponding chloroplast DNA copy number (Extended Data Fig. 11). In hexaploid wheat, a complete chloroplast genome assembly was achievable at a WGS depth of ≥ 0.02 ×. However, the required sequencing depth varied across species due to differences in chloroplast copy number [7]. The relatively high chloroplast DNA copy number in hexaploid wheat enabled successful assembly at lower WGS depths. These findings suggest that leveraging nuclear-to-plastid copy number differences can facilitate the rapid and efficient assembly of chloroplast genomes.

### Chloroplast inheritance in hexaploid wheat

Allopolyploidy refers to the formation of new species through hybridization and polyploidization of different species. The hybrid species formed by allopolyploidy contain two distinct genomes, but their organellar genomes typically follow strict maternal inheritance. Therefore, allopolyploids not only face regulatory challenges in gene expression between genomes but must also adapt to nuclear-cytoplasmic interactions to process redundant gene expression patterns, imposing significant genetic pressure on these plants. This study developed a model suitable for investigating the inheritance pattern of chloroplasts in allopolyploid plants. After obtaining hexaploid wheat and its wild relatives, a genetic variation map of the wheat chloroplast was constructed. The results show that the genetic distance of the hexaploid wheat chloroplast is very close to that of the tetraploid wheat (*F*_ST_ = 0.014) and far from that of *Aegilops tauschii* (*F*_ST_ = 0.62). PCA and population structure analysis further confirmed that the chloroplast of hexaploid wheat was inherited from tetraploid wheat. Additionally, the chloroplast genetic diversity of hexaploid wheat is lower than that of its two ancestral species, further proving that the chloroplast of hexaploid wheat is not inherited from both ancestral species. Thus, it is concluded that the maternal donor of hexaploid wheat is tetraploid wheat, while *Aegilops tauschii* serves as the paternal donor, a role that remains consistent throughout the formation of hexaploid wheat. The formation of allopolyploid species often involves hybridization and polyploidization in a fixed manner that retains the genomic contributions of both parental species. This process is likely an optimal strategy to minimize nucleocytoplasmic disturbances during allopolyploidization. In hexaploid wheat, the proportional expansion of the nuclear genome leads to an increase in cell size, which necessitates a corresponding elevation in gene expression. Concurrently, we observed the rise in chloroplast DNA copy number in hexaploid wheat (Fig. 2A), which was significantly greater than that observed in tetraploid wheat and likely to meet the functional demands of maintaining normal cellular processes. Based on these observations, we hypothesize that dosage effects driven by increased genome copy numbers may represent a key mechanism underlying nucleocytoplasmic interactions between *Triticum* and *Aegilops* species.

### Frequent intra-recombination in the IR regions

Similar to the nuclear genome, the chloroplast genome needs to maintain its stability to avoid the emergence of potentially harmful phenotypes. In this study, conflicts in the direction of single-copy sequences were observed in the assembled chloroplast genomes, and these direction conflicts were found to be random. Additionally, signals of LD decay were detected in the IR region, suggesting that recombination may occur in this region. The presence of two directions of single-copy sequences in the sequencing data of a single sample, with a near 1:1 ratio, indicates frequent recombination in the chloroplast IR region and supports the idea of two equimolar isoforms. But observed in *Pinaceae*, the coexistence of both genomic isomers within individual cupressophyte plants remains uncertain due to homologous recombination at intermediately sized IRs [19]. Studies by Schardl, Feng, and others [14, 49] have shown that organellar genome sequence recombination is not limited to large segmental repeats, as microhomologous sequences can also mediate recombination events. Based on these findings and considering the accumulation of numerous indel-type rare variations in the chloroplast IR region, we hypothesize that the high proportion of indels in the chloroplast may be related to the frequent recombination events, representing sequence changes resulting from recombination. In this study, we also demonstrated that the occurrence of indels in the LSC region is more strongly correlated with short repeat sequences than SNPs, being 1.6 times higher than SNPs.

### Diversity and conservation of intra-recombination in the IR regions

Whether intramolecular recombination occurs within the IR regions of chloroplast genomes remains a central issue in the study of chloroplast genome evolution. Because of the evolutionary patterns between different structural regions of the chloroplast genome vary. Our findings demonstrate that recombination generates two configurations only introduce 1–2 bp indels at the recombination sites, leading to higher diversity in the IR region compared to other single-copy regions. On the contrary, in grasses, the diversity in the IR region is lower than that in the SC region. This may be because the IR region typically contains highly conserved genes (such as rRNA genes), and functional constraints limit the diversity of this region through purifying selection. In this study, Tajima's D was calculated for different genomic structures, and the results support the aforementioned analysis. Although the chloroplast genome as a whole is under negative selection, the IR region experiences stronger purifying selection than the SC region. Despite the greater effect of purifying selection in the IR region, recombination within the IR region increases diversity within populations (the chloroplast IR region has higher diversity than the SC region in the wheat population), which helps the population adapt to local environmental changes, eliminate harmful mutations in the IR region, and maintain the proper functioning of essential genes. Structurally, recombination in the IR region preserves the consistency of two gene sequences, thus preventing gene expression imbalance caused by the accumulation of differences. For example, in leguminous plants, the loss of the IR region leads to an increased mutation rate in that region [35, 38], highlighting the role of the IR in maintaining the stability of the chloroplast genome. Therefore, our study provides an explanation for the maintenance of consistency in chloroplast IR sequences during evolution. The low diversity of the IR region between species indicates that its function is under strict constraints, while recombination allows sufficient genetic flexibility to be maintained within specific regions. Although, the underlying mechanisms remain unclear, this balance is crucial for long-term evolutionary processes.

## Conclusion

In this study, we assembled and annotated the chloroplast genomes of 61 hexaploid wheat and its closely related ancestral species, making them publicly available. These genomes include 28 hexaploid wheat accessions, 24 tetraploid wheat accessions, and 9 *Aegilops tauschii* accessions. A total of 1,298 variant sites were identified to investigate the genetic diversity of wheat chloroplasts and to determine the tetraploid wheat as a maternal donor involved in forming hexaploid wheat. We detected LD decay in the chloroplast IR regions and confirmed the presence of two inversion isomers in equal proportions within individual samples. Given that recombination can introduce indels, we found a higher proportion of indels in the IR regions compared to the SC regions, collectively indicating the occurrence of frequent intramolecular recombination in the IR region of the wheat chloroplast genome. Previously, recombination in the IR region had only been inferred through experimental methods in *Osmunda*. Our findings suggest that frequent intramolecular recombination enhances IR diversity within wheat populations. However, in *Poeceae*, the IR region exhibits lower diversity than the SC region. The contrasting patterns of IR diversity within populations and between species may be attributed to frequent recombination within populations and strong purifying selection acting on the IR region at the interspecies level.

## Methods

### Assembly of wheat chloroplast genome from filtered whole-genome sequences

To represent the genetic diversity of wheat chloroplasts, we selected 61 accessions for HiFi sequencing on the PacBio Sequel II system, that contains 28 bread wheat, 24 tetraploid wheat, and 9 *Aegilops*. Chloroplast genome sequences from phylogenetically diverse plants were selected to identify conserved regions of the wheat chloroplast genome (Supplementary Table 3) Homologous sequence alignment was used to filter sequencing data containing the complete conserved regions of the wheat chloroplast genome, which were then designated as the chloroplast sequencing dataset. The sequencing depth of the filtered chloroplast data was correlated with the copy number of chloroplasts in leaf cells, with an average depth exceeding 500 ×, meeting the requirements for chloroplast genome assembly. To verify whether the filtered data originated from the chloroplast, filtered data were mapped to the genome using BWA-MEM [32], and the results were filtered with Q30 by SAMtools (v1.7) [33]. The de novo assembly was conducted using HiCanu v2.0 [41] with the parameters ‘genomeSize = 135 k contigFilter = "2 0 1.0 0.5 0" readSamplingCoverage = 120 readSamplingBias = 1000 -pacbio-hifi’. In the raw data, the classification of IRa and IRb in this study was determined based on their relative sequence orientation (Fig. 1A). Two pseudo-genomes were constructed, each centered on IRa or IRb and extending 2 kb upstream and downstream, resulting in two pseudo-genomes, each 25.5 kb in length. Sequencing data for the two IRs were classified and assembled separately. Finally, the quadripartite structure of the chloroplast genome was manually reconstructed and completed.

### Construction of the wheat chloroplast pan-genome and variation map

In this study, 61 chloroplast genomes from wheat and its closely related ancestral species were assembled and annotated. The chloroplast pan-genome of wheat was constructed using whole-genome sequence alignment performed with MAFFT (v7.407) [26]. Considering the high proportion of indels in chloroplast genomes among species and the lack of suitable software for organellar variant detection, this study utilized whole-genome alignment to identify all variation sites among samples. We first aligned the chloroplast genome sequences using MAFFT v7.407 and then extract SNP positions from the multiple sequence alignment. Variations greater than 50 bp were classified as SV. The following formula was used to calculate *π* [40]. *π* = D/L/(N × (N − 1)/2), where D represents the number of differential sites, L represents the length of the conserved alignment and N represents the number of sequences.

### Phylogeny and population structure analysis

A total of 1,300 high-quality variation sites were identified, including 650 SNPs, 646 indels, and 2 structural variants. The phylogenetic tree was reconstructed using RAxML software (v8.2.12) [51], with a 1000-times bootstrap in the GTRGAMMA model, and the output tree was plotted in iTOL [31]. The parameters were ‘-T 10 -f a -m GTRGAMMA -p 12,345—× 12,345 -# 1000’. To compare the diversity among different ploidy-level subpopulations, we calculated the nucleotide diversity for each subpopulation across the genome in a window of 1 kb. We initially performed a population structure analysis using the ADMIXTURE (v1.3.0) [1] software, with *k* ranging from 2 to 7. Here *k* = 2 was subsequently chosen because it was the minimal value of *k*. The principal components analysis (PCA) and Weir and Cockerham’s *F*_ST_ value per site between two populations was calculated using VCFtools (0.1.15) [12]. To estimate the mean *F*_ST_ value between two populations, we averaged *F*_ST_ values calculated from all SNPs.

### Recombination landscapes

All the polymorphic markers used in this research are identified SNP and indel markers, we calculated LD across the genome using PLINK [50]. We conducted statistical associations between mutations for the entire chloroplast genome as well as for different genomic structures separately. To more intuitively detect recombination events in the IR region, two pseudo-reference genomes were constructed, each centered on the IR region and extended 2 kb to both ends to form a 25.5 kb reference sequence. The difference between the two pseudo-genomes lies in the polarity of their connections to the SC regions (Fig. 5). A search and count were performed in the chloroplast sequencing dataset to represent the support for the two configurations resulting from recombination.

### Chloroplast genome selection analysis

We utilized Tajima’s D to calculate the selection effects on different structures of the chloroplast genome, using the formula [54]:

$$D=\frac{\pi - \theta s}{\surd \text{Var}(\pi - \theta s)}$$

For genome-wide sliding window analysis, Tajima's D was calculated independently for each population. In the wheat chloroplast genome, a sliding window of 1 kb with a step size of 0.5 kb was used. Because adjacent windows overlap, in the genome browser track, Tajima's D is reported for each window using the coordinates of the central 10 kb. A high Tajima's D value indicates an excess of common variants in a region, which may be consistent with balancing selection or population contraction. Conversely, a negative Tajima's D value suggests an excess of rare variants, which aligns with population expansion or positive selection. The theoretical distribution of Tajima's D assumes that polymorphism ascertainment is independent of allele frequency. The distribution of the allele frequency spectrum of the chloroplast genome was combined to infer the selection pressures experienced by different genomic structures of the chloroplast during evolution.

### SSRs and repeat sequences analysis

For the SSRs and repeat sequences, we utilized the k-mer method. Sliding windows of different k-mer lengths were used to calculate the frequency of each repeat sequence. We selected k-mers ranging from 3 to 30 bp in length for analysis. To account for shorter k-mers being nested within longer k-mers, we removed shorter k-mers before recalculating their frequencies. Additionally, to ensure that identical k-mer sequences do not overlap, we ensured their separation to maintain the possibility of recombination occurring in the sequence.

## Supplementary Information

Additional file 1: Supplementary Fig.1. Quality values of chloroplast sequencing data. Supplementary Fig.2. Re-mapping of sequencing data to the assembled genome. Supplementary Fig.3. Distribution of heterozygous variant sites detected across samples. Supplementary Fig.4. Variation map of wheat chloroplast genome. Supplementary Fig.5. Intramolecular recombination in chloroplast genome. Supplementary Fig.6. Dot plot of LSC and SSC direction in contigs. Supplementary Fig.7. Allele frequency distribution of indels and SNPs in hexaploid wheat (N = 28). Supplementary Fig.8. Distribution of SNP in IRa and IRb. Supplementary Fig.9. Tajima’s D statistic in subpopulations. Supplementary Fig.10. GC content in wheat chloroplast genome. Supplementary Fig.11. Differences in chloroplast genome assembly sequences from sequencing data of varying depths

Additional file 2: Supplementary Table.1.Classification of hexaploid wheat and its parental donor relatives in this study. Supplementary Table.2. Passport information of all accessions in this study. Supplementary Table.3. Chloroplast reference genome information for seven species. Supplementary Table.4. Whole genome sequence depth and chloroplast depth in wheat. Supplementary Table.5. Mapping rate of chloroplast sequencing data. Supplementary Table.6. Genome assembly information of all accessions in this study. Supplementary Table.7. Copy number per leaf cell of chloroplast of all accessions in this study. Supplementary Table.8. CV error of each K values. Supplementary Table.9. Number of pairwise measures of R^2^.

## Data Availability

A portion of the raw sequence data was deposited in the Genome Sequence Archive (GSA) (https://ngdc.cncb.ac.cn/gsa/) under accession numbers PRJCA005995, including diploid, tetraploid and hexaploid samples of wheat genus, with a total of three publicly available raw sequence datasets. The chloroplast sequencing data filtered from the raw reads have been deposited in Zenodo and linked to the corresponding GitHub repository. In addition, the 61 assembled and annotated chloroplast genomes of wheat and its ancestral relatives are also available on GitHub at: https://github.com/Hua-Zhang1339/Chloroplast-genome-analysis.
