# Supplementary material for: Wheat chloroplast pangenome reveals frequent intramolecular recombination in the inverted repeat regions
Source: BMC Plant Biol. 2025 Nov 27;25:1654. doi: 10.1186/s12870-025-07577-5 (PMC12659086; doi:10.1186/s12870-025-07577-5)
Supplement: Supplementary file 1 — Additional file 1: Supplementary Fig.1. Quality values of chloroplast sequencing data. Supplementary Fig.2. Re-mapping of sequencing data to the assembled genome. Supplementary Fig.3. Distribution of heterozygous variant sites detected across samples. Supplementary Fig.4. Variation map of wheat chloroplast genome. Supplementary Fig.5. Intramolecular recombination in chloroplast genome. Supplementary Fig.6. Dot plot of LSC and SSC direction in contigs. Supplementary Fig.7. Allele frequency distribution of indels and SNPs in hexaploid wheat (N = 28). Supplementary Fig.8. Distribution of SNP in IRa and IRb. Supplementary Fig.9. Tajima’s D statistic in subpopulations. Supplementary Fig.10. GC content in wheat chloroplast genome. Supplementary Fig.11. Differences in chloroplast genome assembly sequences from sequencing data of varying depths [file 12870_2025_7577_MOESM1_ESM.pdf]

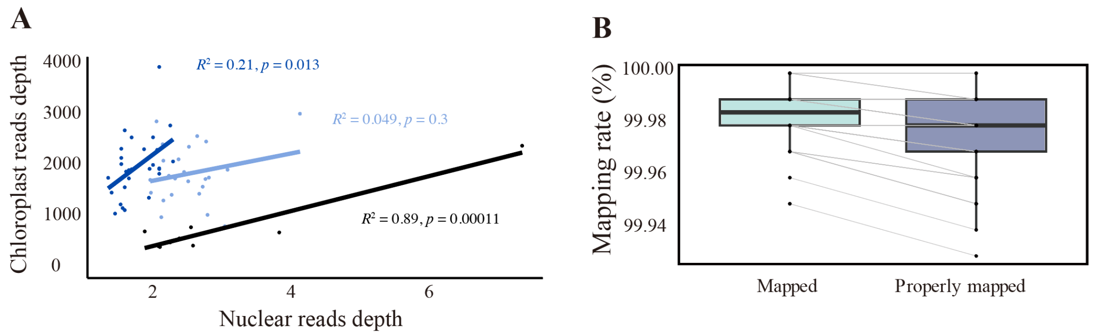

Extended Data Fig. 1 | Quality values of chloroplast sequencing data. (A) Dot plot showing the read depths for nuclear and chloroplast genomes. Dark blue represents hexaploid wheat, light blue represents tetraploid wheat, and black represents *Aegilops*. (B) Mapping rates of re-captured chloroplast reads. The boxplot displays the relationship between the mapped rate and properly mapped rate across all samples. The properly mapped rate of re-captured chloroplast reads exceeds 99%.

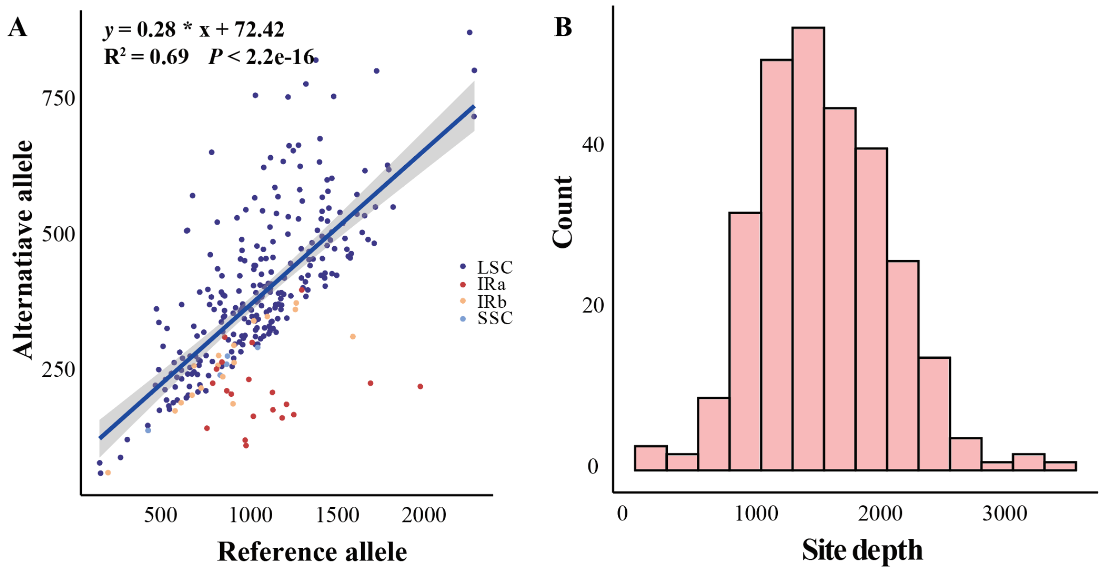

Extended Data Fig. 2 | Re-mapping of sequencing data to the assembled genome. (A) Scatter plot showing variant sites where the sequencing data differ from the assembled genome. The x-axis represents the reference allele, and the y-axis represents the alternative allele. (B) Histogram depicting the coverage depth at each heterozygous site.

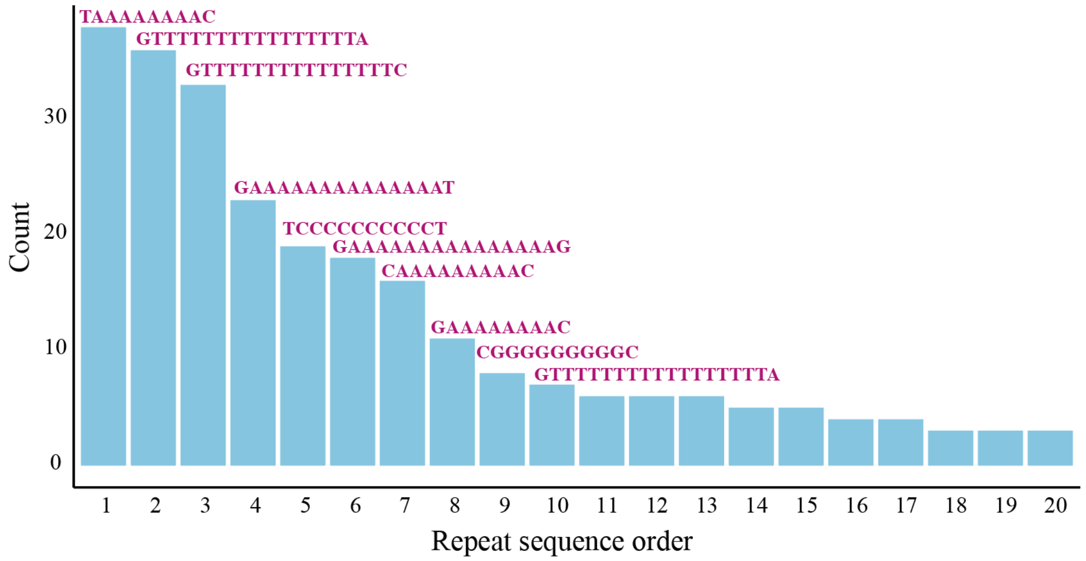

Extended Data Fig. 3 | Distribution of heterozygous variant sites detected across samples. All heterozygous variants are indels, which are shared among different samples. For example, the indel **TAAAAAAAAC** is present as a heterozygous site in more than 30 samples.

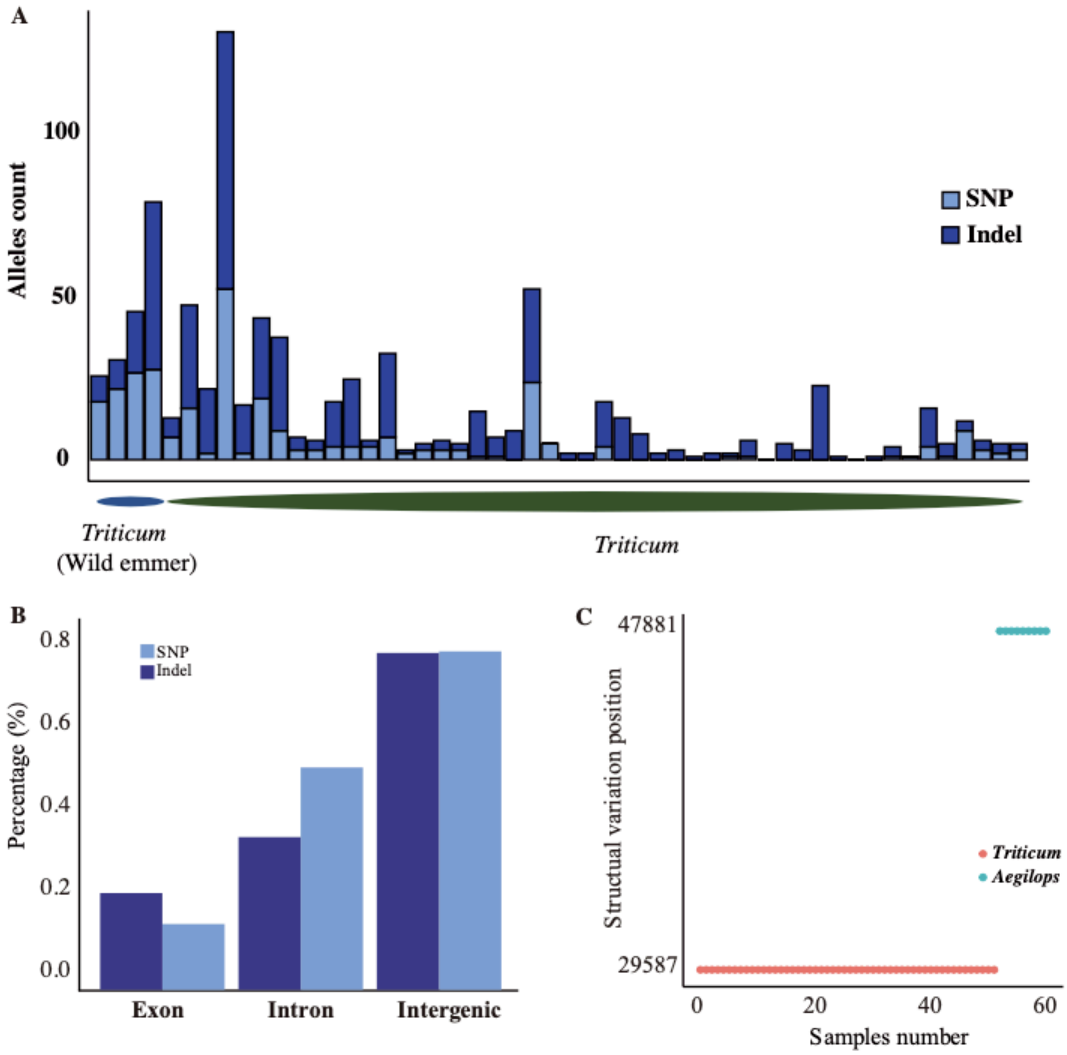

Extended Data Fig. 4 | Variation map of wheat chloroplast genome. (A) The count of SNP and Indel of per accession in *Triticum*. (B) The distribution of SNP and Indel in chloroplast genome structure. (C) Structure variation in *Triticum* and *Aegilops*.

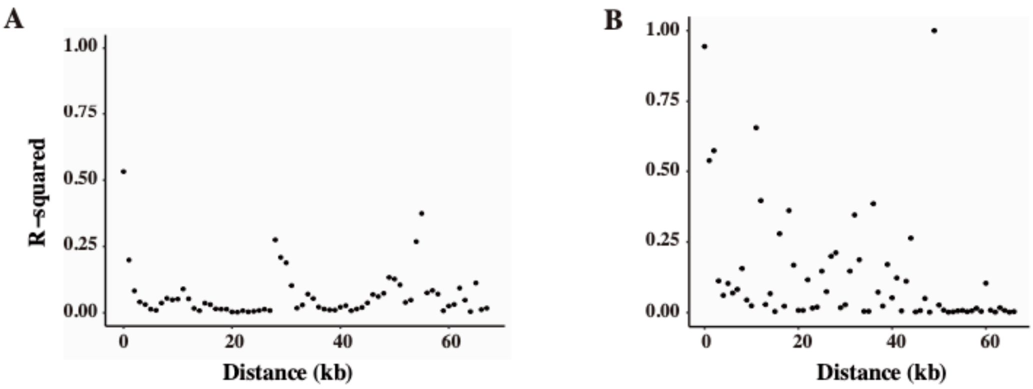

Extended Data Fig. 5 | Intramolecular recombination in chloroplast genome. (A) LD decay in whole chloroplast genome. (B) LD decay in LSC in chloroplast genome.

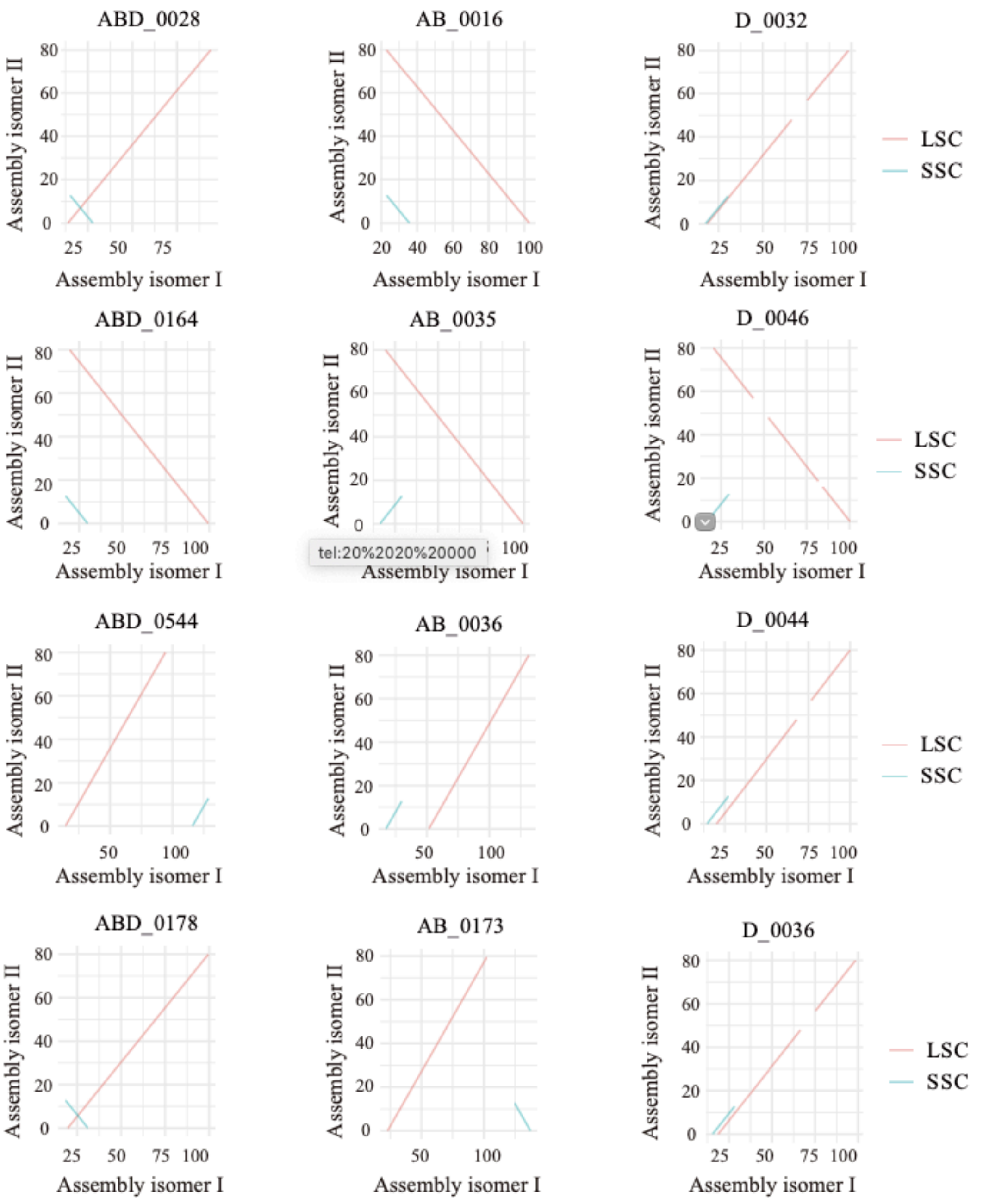

Extended Data Fig. 6 | Dot plot of LSC and SSC direction in contigs. The SC direction of accessions with different ploidy levels exhibits randomness.

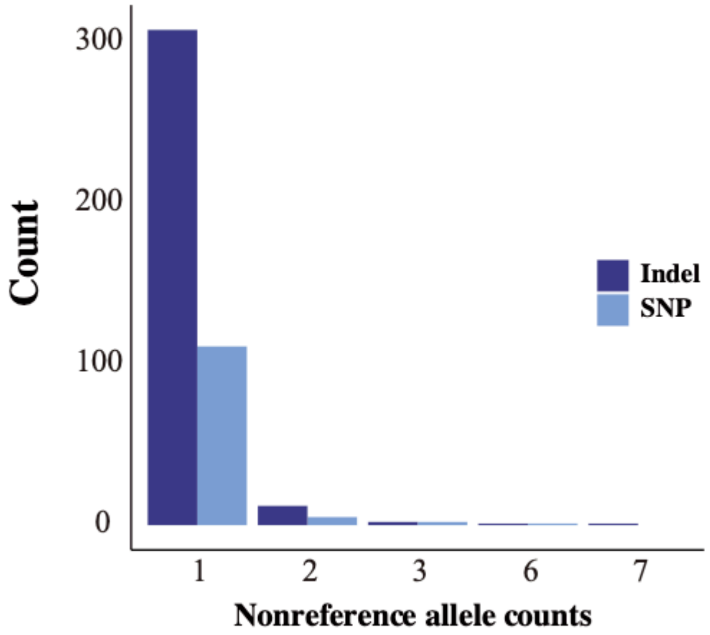

Extended Data Fig. 7 | Allele frequency distribution of indels and SNPs in hexaploid wheat (N = 28).

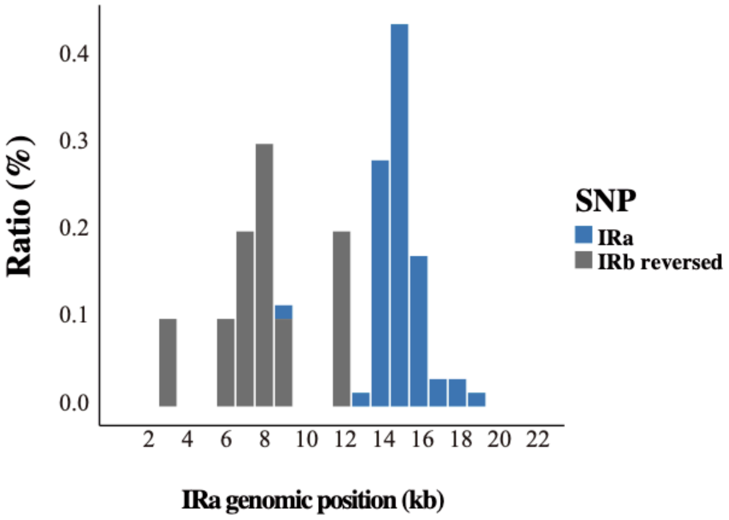

Extended Data Fig. 8 | Distribution of SNP in IRa and IRb. In the reference genome, the position of IRa is marked in blue, while gray represents the reversed sequencing of IRb to ensure consistency with the sequence of IRa.

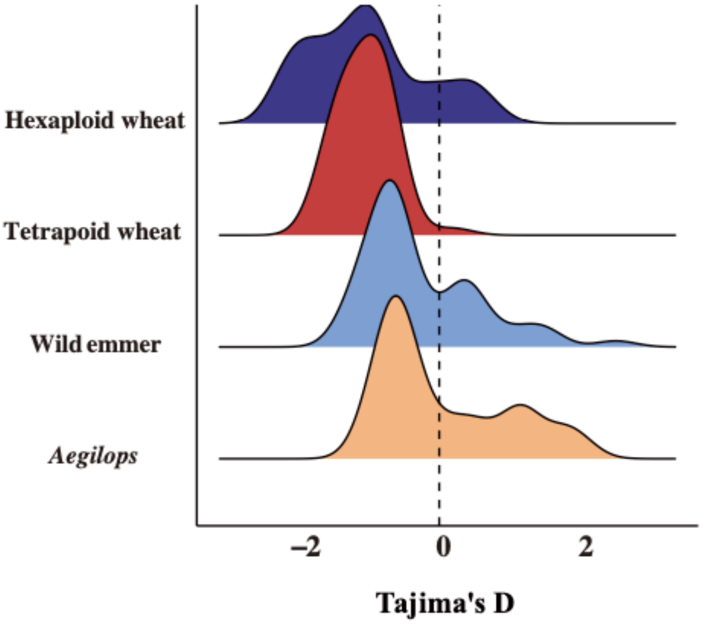

Extended Data Fig. 9 | Tajima’s D statistic in subpopulations.

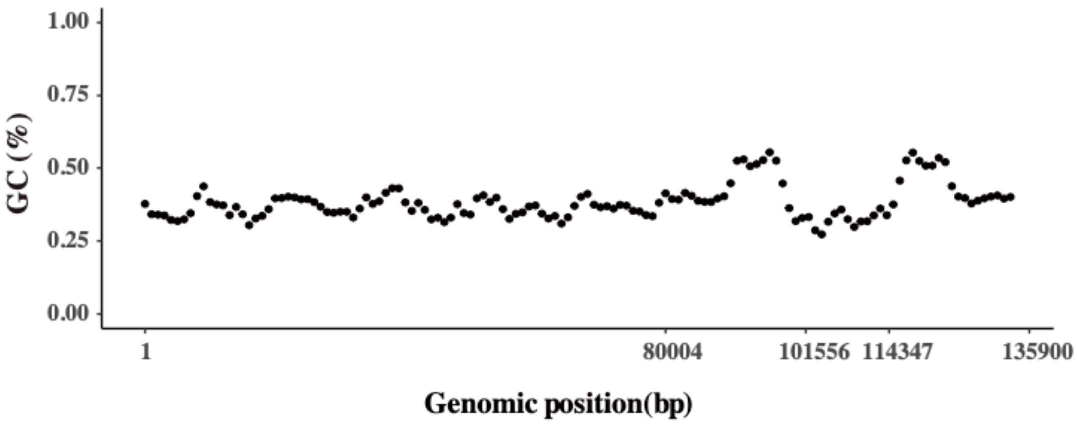

Extended Data Fig. 10 | GC content in wheat chloroplast genome

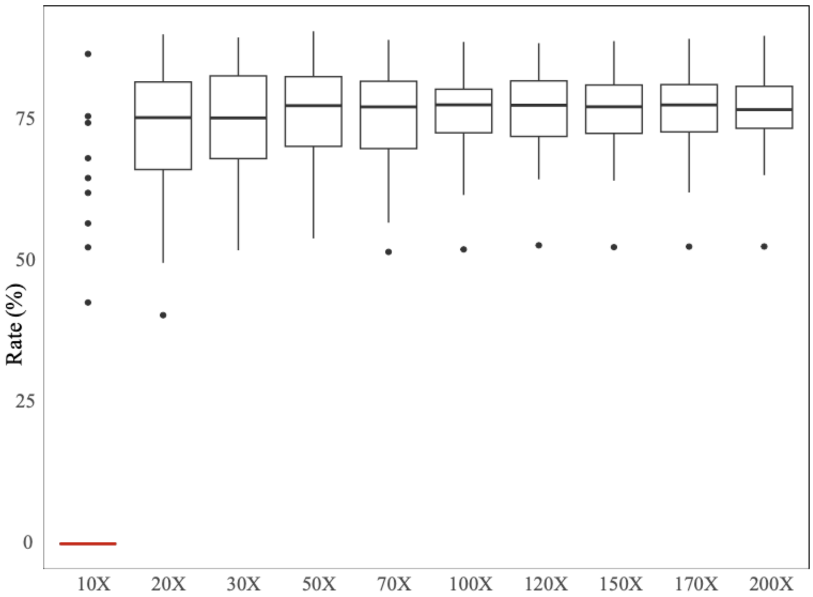

Extended Data Fig. 11 | Differences in chloroplast genome assembly sequences from sequencing data of varying depths.
