# Supplementary material for: Wheat chloroplast pangenome reveals frequent intramolecular recombination in the inverted repeat regions
Source: BMC Plant Biol. 2025 Nov 27;25:1654. doi: 10.1186/s12870-025-07577-5 (PMC12659086; doi:10.1186/s12870-025-07577-5)
Supplement: Supplementary file 2 — Additional file 2: Supplementary Table.1.Classification of hexaploid wheat and its parental donor relatives in this study. Supplementary Table.2. Passport information of all accessions in this study. Supplementary Table.3. Chloroplast reference genome information for seven species. Supplementary Table.4. Whole genome sequence depth and chloroplast depth in wheat. Supplementary Table.5. Mapping rate of chloroplast sequencing data. Supplementary Table.6. Genome assembly information of all accessions in this study. Supplementary Table.7. Copy number per leaf cell of chloroplast of all accessions in this study. Supplementary Table.8. CV error of each K values. Supplementary Table.9. Number of pairwise measures of R2. [file 12870_2025_7577_MOESM2_ESM.pdf]

Supplementary Table 1: Classification of hexaploid wheat and its parental donor relatives in this study

Ploidy	Genome type	Species	Common name	Count
Hexaploid	AABBDD	*Triticum aestivum*	Bread wheat	28
Tetraploid	AABB	*Triticum turgidum*	Tetraploid wheat	24
Diploid	DD	*Aegilops tauschii*	Strangulata	9

Note: In this study, whole-genome sequencing was performed on 61 samples, including three different ploidy levels of bread wheat and their ancestral relatives.

Supplementary Table 2: Passport information of all accessions in this study

SampleID	Accession ID	Subspecies	Country	Common name
AB_0001	PI341300	Triticum turgidum L. subsp. turgidum	Turkey	Free_threshing_tetraploid
AB_0016	CItr14892	Triticum turgidum L. subsp. polonicum (L.) Thell.	Ethiopia	Free_threshing_tetraploid
AB_0023	PI466933	Triticum turgidum L. subsp. dicoccoides (Korn. ex Asch. & Graebn.) Thell.	Syria	Wild_emmer
AB_0024	TRI11505	Triticum turgidum L. subsp. dicoccoides (Korn. ex Asch. & Graebn.) Thell.	Lebanon	Wild_emmer
AB_0027	PI487260	Triticum turgidum L. subsp. dicoccoides (Korn. ex Asch. & Graebn.) Thell.	Syria	Wild_emmer
AB_0031	PI428071	Triticum turgidum L. subsp. dicoccoides (Korn. ex Asch. & Graebn.) Thell.	Turkey	Wild_emmer
AB_0035	PI466941	Triticum turgidum L. subsp. dicoccoides (Korn. ex Asch. & Graebn.) Thell.	Syria	Wild_emmer
AB_0036	PI272602	Triticum turgidum L. subsp. turanicum (Jakubz.) A. Love & D. Love	Hungary	Free_threshing_tetraploid
AB_0037	PI254192	Triticum turgidum L. subsp. dicoccon (Schrank) Thell.	Serbia	Domesticated_emmer
AB_0038	PI626391	Triticum turgidum L. subsp. dicoccon (Schrank) Thell.	Iran	Domesticated_emmer
AB_0052	PI624908	Triticum turgidum L. subsp. dicoccon (Schrank) Thell.	Iran	Domesticated_emmer
AB_0054	PI499973	Triticum turgidum L. subsp. dicoccon (Schrank) Thell.	Armenia	Domesticated_emmer
AB_0072	PI624904	Triticum turgidum L. subsp. dicoccon (Schrank) Thell.	Iran	Domesticated_emmer
AB_0081	PI94752	Triticum turgidum L. subsp. carthlicum (Nevski) A. Love & D. Love	Georgia	Free_threshing_tetraploid
AB_0089	PI352493	Triticum ispahanicum Heslot	Iran	OtherTetraploid
AB_0090	TRI11946	Triticum karamyschevii NEVSKI var. karamyschevii	Georgia	OtherTetraploid
AB_0091	PI178143	Triticum turgidum L. subsp. durum (Desf.) Husn.	Turkey	Free_threshing_tetraploid
AB_0097	PI627514	Triticum turgidum L. subsp. durum (Desf.) Husn.	Iran	Free_threshing_tetraploid
AB_0104	PI32877	Triticum turgidum L. subsp. durum (Desf.) Husn.	Kazakhstan	Free_threshing_tetraploid
AB_0111	PI428016	Triticum turgidum L. subsp. dicoccoides (Korn. ex Asch. & Graebn.) Thell.	Iran	Wild_emmer
AB_0113	PI487254	Triticum turgidum L. subsp. dicoccoides (Korn. ex Asch. & Graebn.) Thell.	Syria	Wild_emmer
AB_0116	PI428098	Triticum turgidum L. subsp. dicoccoides (Korn. ex Asch. & Graebn.) Thell.	Israel	Wild_emmer
AB_0122	PI470998	Triticum turgidum L. subsp. dicoccoides (Korn. ex Asch. & Graebn.) Thell.	Israel	Wild_emmer
AB_0173	PI428066	Triticum turgidum L. subsp. dicoccoides (Korn. ex Asch. & Graebn.) Thell.	Turkey	Wild_emmer
ABD_0028	PI337997	Triticum aestivum L. subsp. sphaerococcum (Percival) MacKey	India	OtherHexaploid
ABD_0034	XM0920	Triticum aestivum ssp.yunna-nense King	China	Landrace
ABD_0059	PI184157	Triticum aestivum L. subsp. aestivum	BHI	Landrace
ABD_0085	PI157949	Triticum aestivum L. subsp. aestivum	Italy	Landrace
ABD_0162	HongChunMai	Triticum aestivum L. subsp. aestivum	China	Landrace
ABD_0164	HanZhongBai	Triticum aestivum L. subsp. aestivum	China	Landrace
ABD_0178	PI94430	Triticum aestivum L. subsp. aestivum	Belarus	Landrace
ABD_0181	PI195415	Triticum aestivum L. subsp. aestivum	Guatemala	Landrace
ABD_0235	PI192206	Triticum aestivum L. subsp. aestivum	Portugal	Landrace
ABD_0240	PI196078	Triticum aestivum L. subsp. aestivum	Ethiopia	Landrace
ABD_0260	PI350727	Triticum aestivum L. subsp. Aestivum	Austria	Landrace
ABD_0261	PI350744	Triticum aestivum L. subsp. aestivum	Austria	Landrace
ABD_0283	PI436202	Triticum aestivum L. subsp. aestivum	Chile	Landrace
ABD_0295	PI477878	Triticum aestivum L. subsp. aestivum	Peru	Landrace
ABD_0301	PI565223	Triticum aestivum L. subsp. aestivum	Bolivia	Landrace
ABD_0335	PI94520	Triticum aestivum L. subsp. aestivum	Azerbaijan	Landrace
ABD_0338	PI572687	Triticum aestivum L. subsp. aestivum	Georgia	Landrace
ABD_0341	PI182665	Triticum aestivum L. subsp. aestivum	Lebanon	Landrace

Supplementary Table 2: Passport information of all accessions in this study (Continued table)

SampleID	Accession ID	Subspecies	Country	Common name
ABD_0356	PI178212	Triticum aestivum L. subsp. aestivum	Iraq	Landrace
ABD_0357	PI178756	Triticum aestivum L. subsp. aestivum	Iraq	Landrace
ABD_0435	兰考 86(79)1-2-8-1	Triticum aestivum L. subsp. aestivum	China	Cultivar
ABD_0451	周麦 16	Triticum aestivum L. subsp. aestivum	China	Cultivar
ABD_0467	邯麦 13	Triticum aestivum L. subsp. aestivum	China	Cultivar
ABD_0477	皖麦 19	Triticum aestivum L. subsp. aestivum	China	Cultivar
ABD_0506	绵阳 26 号	Triticum aestivum L. subsp. aestivum	China	Cultivar
ABD_0535	晋农 207	Triticum aestivum L. subsp. aestivum	China	Cultivar
ABD_0544	Ponderosa	Triticum aestivum L. subsp. aestivum	China	Cultivar
CS				
D0014	PI 560237	Aegilops tauschii Coss.	Azerbaijan	Ae.tauschii
D0015	PI560755	Aegilops tauschii Coss.	Turkey	Ae.tauschii
D0022	PI 603234	Aegilops tauschii Coss.	Azerbaijan	Ae.tauschii
D0032	TA 2377	Aegilops tauschii Coss.	Iran	Ae.tauschii
D0036	TA 2453	Aegilops tauschii f. strangulata	Iran	Ae.tauschii
D0046	AE 1211	Aegilops tauschii f. strangulata	Armenia	Ae.tauschii
D0047	AE 1600	Aegilops tauschii Coss. subsp. strangulata (Eig) Tzvelev	Iran	Ae.tauschii
D0048	AE 1602	Aegilops tauschii Coss. subsp. strangulata (Eig) Tzvelev	Iran	Ae.tauschii
D0044	TA 2470	Aegilops tauschii Coss. subsp. strangulata (Eig) Tzvelev	Iran	Ae.tauschii

Supplementary Table 3: Chloroplast reference genome information for seven species

Species	Genome type	NCBI reference sequence	Length
Triticum aestivum	Chloroplast	NC_002762.1	134,545
Hordeum vulgare	Chloroplast	NC_008590.1	136,462
Sorghum bicolor	Chloroplast	NC_008602.1	140,754
Agrostis stolonifera	Chloroplast	NC_008591.1	136,584
Oryza sativa	Chloroplast	NC_001320.1	134,525
Zea mays	Chloroplast	NC_001666.2	140,384
Phalaenopsis aphrodite	Chloroplast	NC_007499.1	148,964

Note: The table provides the species names, lengths, and NCBI accession numbers of the chloroplast reference genomes for seven species

Supplementary Table 4: Whole genome sequence depth and chloroplast depth in wheat

SampleID	Whole genome sequence reads size (Gb)	Wheat whole genome reference size (Gb)	Whole genome sequence depth	Filtered chloroplast sequence reads size (Mb)	Wheat chloroplast genome reference (Kb)	Chloroplast genome sequence depth
AB_0001	24.2	10	2.42	294.4	136	2164.7
AB_0016	28.2	10	2.82	341.9	136	2514.0
AB_0023	27.7	10	2.77	221.2	136	1626.5
AB_0024	22.0	10	2.20	290.8	136	2138.2
AB_0027	28.4	10	2.84	146.0	136	1073.5
AB_0031	21.7	10	2.17	139.8	136	1027.9
AB_0035	22.0	10	2.20	184.4	136	1355.9
AB_0036	27.2	10	2.72	202.4	136	1488.2
AB_0037	20.2	10	2.02	205.1	136	1508.1
AB_0038	41.8	10	4.18	416.2	136	3060.3
AB_0052	28.6	10	2.86	245.6	136	1805.9
AB_0054	31.3	10	3.13	266.6	136	1960.3
AB_0072	30.9	10	3.09	115.6	136	850.0
AB_0081	21.0	10	2.10	395.8	136	2910.3
AB_0089	25.7	10	2.57	327.9	136	2411.0
AB_0090	23.2	10	2.32	199.6	136	1467.6
AB_0091	26.9	10	2.69	356.5	136	2621.3
AB_0097	23.7	10	2.37	243.1	136	1787.5
AB_0104	22.8	10	2.28	250.4	136	1841.2
AB_0111	25.6	10	2.56	235.6	136	1732.4
AB_0113	21.9	10	2.19	334.7	136	2461.0
AB_0116	20.1	10	2.01	239.3	136	1759.6
AB_0122	28.1	10	2.81	241.8	136	1777.9
AB_0173	25.0	10	2.50	260.5	136	1915.4
ABD_0028	30.0	14	2.14	541.2	136	3979.4
ABD_0034	22.2	14	1.59	298.1	136	2191.9
ABD_0059	22.9	14	1.64	158.7	136	1166.9
ABD_0085	24.4	14	1.74	352.9	136	2594.9
ABD_0162	29.5	14	2.11	319.6	136	2350.0
ABD_0164	22.5	14	1.61	164.1	136	1206.6
ABD_0178	28.7	14	2.05	279.4	136	2054.4
ABD_0181	30.0	14	2.14	270.4	136	1988.2
ABD_0235	23.2	14	1.66	261.6	136	1923.5
ABD_0240	28.7	14	2.05	353.2	136	2597.1
ABD_0260	27.9	14	1.99	191.7	136	1409.6
ABD_0261	32.8	14	2.34	288.6	136	2122.1
ABD_0283	21.0	14	1.50	149.4	136	1098.5
ABD_0295	30.0	14	2.14	255.8	136	1880.9
ABD_0301	24.3	14	1.74	267.9	136	1969.6
ABD_0335	23.8	14	1.70	243.5	136	1790.4
ABD_0338	22.1	14	1.58	173.9	136	1278.7

Supplementary Table 4: Whole genome sequence depth and chloroplast depth in wheat (Continued table)

SampleID	Whole genome sequence reads size (Gb)	Wheat whole genome reference size (Gb)	Whole genome sequence depth	Filtered chloroplast sequence reads size (Mb)	Wheat chloroplast genome reference (Kb)	Chloroplast genome sequence depth
ABD_0341	30.0	14	2.14	268.5	136	1974.3
ABD_0356	28.0	14	2.00	269.0	136	1977.9
ABD_0357	19.6	14	1.40	244.9	136	1800.7
ABD_0435	22.9	14	1.64	219.8	136	1616.2
ABD_0451	20.3	14	1.45	205.5	136	1511.0
ABD_0467	22.2	14	1.59	281.7	136	2071.3
ABD_0477	22.2	14	1.59	322.6	136	2372.1
ABD_0506	23.8	14	1.70	265.5	136	1952.2
ABD_0535	26.9	14	1.92	321.4	136	2363.2
ABD_0544	23.0	14	1.64	371.8	136	2733.8
CS	32.2	14	2.30	384.8	136	2829.4
D0014	9.2	4	2.30	72.6	136	533.8
D0015	8.5	4	2.13	62.3	136	458.1
D0022	7.7	4	1.93	101.8	136	748.5
D0032	9.7	4	2.43	83.1	136	611.0
D0036	10.4	4	2.60	112.9	136	830.1
D0046	10.5	4	2.63	63.9	136	469.9
D0047	8.6	4	2.15	60.2	136	442.6
D0048	15.5	4	3.88	99.0	136	727.9
D0044	29.6	4	7.40	330.7	136	2431.6

Supplementary Table 5: Mapping rate of chloroplast sequencing data

SampleID	Mapped reads count	Unmapped reads count	Mapped ratio	Properly mapped ratio	Coverage depth
AB_0001	23049	0	99.99%	99.98%	2164.267
AB_0016	25995	0	99.98%	99.97%	2513.246
AB_0023	18669	0	99.98%	99.98%	1626.175
AB_0024	34732	0	99.99%	99.99%	2137.986
AB_0027	13177	0	99.95%	99.93%	1072.749
AB_0031	12135	0	99.98%	99.96%	1027.489
AB_0035	17930	0	99.99%	99.99%	1355.764
AB_0036	23339	0	99.98%	99.97%	1487.754
AB_0037	19371	0	99.99%	99.98%	1507.798
AB_0038	34755	0	100.00%	100.00%	3060.3
AB_0052	24623	0	99.98%	99.98%	1805.539
AB_0054	21628	0	99.97%	99.95%	1959.32
AB_0072	10276	0	99.96%	99.94%	849.49
AB_0081	36827	0	99.98%	99.98%	2909.718
AB_0089	27595	0	99.97%	99.96%	2410.036
AB_0090	18099	0	99.98%	99.96%	1467.013
AB_0091	29736	0	99.98%	99.97%	2620.514
AB_0097	23054	0	99.98%	99.97%	1786.964
AB_0104	21754	0	99.97%	99.96%	1840.464
AB_0111	19989	0	99.99%	99.99%	1732.227
AB_0113	26726	0	100.00%	99.99%	2460.754
AB_0116	20467	0	99.99%	99.99%	1759.424
AB_0122	21014	0	99.99%	99.99%	1777.722
AB_0173	22227	0	100.00%	99.99%	1915.208
ABD_0028	56978	0	99.99%	99.99%	3979.002
ABD_0034	34361	0	99.99%	99.98%	2191.462
ABD_0059	18489	0	99.98%	99.97%	1166.55
ABD_0085	37724	0	99.99%	99.99%	2594.641
ABD_0162	27425	0	99.97%	99.95%	2348.825
ABD_0164	15764	0	99.95%	99.92%	1205.635
ABD_0178	29429	0	99.99%	99.98%	2053.989
ABD_0181	28433	0	99.98%	99.97%	1987.604
ABD_0235	25182	0	99.98%	99.98%	1923.115
ABD_0240	27342	0	99.99%	99.98%	2596.581
ABD_0260	19954	0	99.99%	99.98%	1409.318
ABD_0261	26525	0	99.98%	99.97%	2121.463
ABD_0283	15645	0	99.99%	99.99%	1098.39
ABD_0295	26451	0	99.99%	99.99%	1880.712

Supplementary Table 5: Mapping rate of chloroplast sequencing data (Continued table)

SampleID	Mapped reads count	Unmapped reads count	Mapped ratio	Properly mapped ratio	Coverage depth
ABD_0301	28596	0	100.00%	99.99%	1969.403
ABD_0335	24248	0	99.99%	99.98%	1790.042
ABD_0338	14824	0	99.98%	99.97%	1278.316
ABD_0341	22785	0	99.98%	99.97%	1973.708
ABD_0356	23855	0	99.99%	99.98%	1977.504
ABD_0357	26421	0	99.98%	99.98%	1800.34
ABD_0435	22299	0	99.99%	99.98%	1615.877
ABD_0451	18829	0	99.98%	99.97%	1510.547
ABD_0467	22394	0	99.99%	99.99%	2071.093
ABD_0477	28388	0	99.97%	99.96%	2371.151
ABD_0506	24190	0	99.98%	99.97%	1951.614
ABD_0535	31399	0	99.99%	99.98%	2362.727
ABD_0544	36806	0	99.99%	99.99%	2733.527
CS	33074	0	99.99%	99.98%	2828.834
D_0014	6552	0	99.98%	99.98%	533.6932
D_0015	5751	0	100.00%	100.00%	458.1
D_0022	9317	0	100.00%	100.00%	748.5
D_0032	8889	0	99.99%	99.98%	610.8778
D_0036	11713	0	99.97%	99.96%	829.768
D_0044	35610	0	99.99%	99.98%	469.806
D_0046	6651	0	99.97%	99.95%	442.3787
D_0047	6193	0	99.97%	99.95%	727.5361
D_0048	10992	0	99.99%	99.98%	2431.114

Note: Mapping numbers, unmapped reads, and coverage depth information for 61 samples.

Supplementary Table 6: Genome assembly information of all accessions in this study

SampleID	Contig number	Minimal contig length	Max contig length	Assembled genome length	Annotation gene count	Assembled genome GC content
AB_0001	7	17,711	147,043	135,899	83	38.32%
AB_0016	1	162,515	162,515	135,899	83	38.31%
AB_0023	5	21,351	115,025	135,949	83	38.31%
AB_0024	6	17,269	115,431	135,896	83	38.31%
AB_0027	1	157,155	157,155	135,936	83	38.32%
AB_0031	1	160,642	160,642	135,982	83	38.31%
AB_0035	7	17,406	116,010	135,900	83	38.31%
AB_0036	7	20,362	119,124	135,891	83	38.32%
AB_0037	2	159,583	166,690	135,901	83	38.31%
AB_0038	3	22,558	119,170	135,937	83	38.31%
AB_0052	3	26,222	118,455	135,895	83	38.31%
AB_0054	2	56,550	130,439	135,900	83	38.32%
AB_0072	6	21,479	156,128	135,900	83	38.32%
AB_0081	4	20,205	152,557	135,906	83	38.31%
AB_0089	2	25,701	156,197	135,913	83	38.32%
AB_0090	1	158,550	158,550	135,901	83	38.32%
AB_0091	5	23,160	152,789	135,956	83	38.33%
AB_0097	3	25,296	158,031	135,898	83	38.32%
AB_0104	3	25,253	158,920	135,899	83	38.32%
AB_0111	6	17,830	164,592	135,898	83	38.32%
AB_0113	2	37,381	164,314	135,904	83	38.31%
AB_0116	1	162,472	162,472	135,922	83	38.32%
AB_0122	2	30,604	171,948	135,901	83	38.32%
AB_0173	2	22,981	159,081	135,984	83	38.31%
ABD_0028	15	14,650	109,512	135,914	83	38.31%
ABD_0034	2	20,364	151,069	135,903	83	38.32%
ABD_0059	4	15,337	119,666	135,900	83	38.32%
ABD_0085	9	15,315	109,114	135,898	83	38.32%
ABD_0162	2	16,881	159,493	135,898	83	38.31%
ABD_0164	3	20,459	116,196	135,902	83	38.32%
ABD_0178	5	17,767	114,501	135,901	83	38.31%
ABD_0181	6	17,160	98,120	135,899	83	38.31%
ABD_0235	5	20,649	121,037	135,900	83	38.32%
ABD_0240	5	23,375	155,434	135,905	83	38.31%
ABD_0260	3	22,304	123,433	135,898	83	38.32%
ABD_0261	2	39,099	162,651	135,895	83	38.32%
ABD_0283	3	29,242	115,089	135,897	83	38.32%
ABD_0295	3	22,877	119,847	135,932	83	38.31%
ABD_0301	5	18,154	114,016	135,904	83	38.32%
ABD_0335	4	20,651	120,599	135,924	83	38.32%
ABD_0338	1	162,680	162,680	135,919	83	38.32%

Supplementary Table 6: Genome assembly information of all accessions in this study (Continued table)

SampleID	Contig number	Minimal contig length	Max contig length	Assembled genome length	Annotation gene count	Assembled genome GC content
ABD_0341	4	27,304	161,743	135,898	83	38.32%
ABD_0356	2	80,581	97,187	135,897	83	38.32%
ABD_0357	4	23,305	117,612	135,900	83	38.32%
ABD_0435	2	23,502	152,363	135,907	83	38.32%
ABD_0451	2	55,493	125,983	135,924	83	38.31%
ABD_0467	3	34,447	180,425	135,918	83	38.32%
ABD_0477	2	33,482	156,915	135,897	83	38.32%
ABD_0506	3	26,882	159,630	135,909	83	38.31%
ABD_0535	2	15,968	160,896	135,913	83	38.31%
ABD_0544	1	159,280	159,280	135,898	83	38.31%
CS	2	23,915	155,597	135,900	83	38.32%
D0014	2	21,225	155,262	135,625	83	38.33%
D0015	2	37,316	141,474	135,558	83	38.33%
D0022	4	20,984	115,960	135,626	83	38.32%
D0032	7	19,943	116,904	135,686	83	38.32%
D0036	2	54,073	116,438	135,606	83	38.33%
D0046	5	19,794	112,683	135,700	83	38.32%
D0047	2	28,666	152,831	135,575	83	38.33%
D0048	9	18,756	118,808	135,698	83	38.32%
D0044	8	19,733	118,145	135,583	83	38.33%

Supplementary Table 7: Copy number per leaf cell of chloroplast of all accessions in this study

SampleID	Whole genome sequence depth	Chloroplast genome sequence depth	Chloroplast copy number per cell
AB_0001	2.42	2164.7	895
AB_0016	2.82	2514.0	891
AB_0023	2.77	1626.5	587
AB_0024	2.20	2138.2	972
AB_0027	2.84	1073.5	378
AB_0031	2.17	1027.9	474
AB_0035	2.20	1355.9	616
AB_0036	2.72	1488.2	547
AB_0037	2.02	1508.1	747
AB_0038	4.18	3060.3	732
AB_0052	2.86	1805.9	631
AB_0054	3.13	1960.3	626
AB_0072	3.09	850.0	275
AB_0081	2.10	2910.3	1,386
AB_0089	2.57	2411.0	938
AB_0090	2.32	1467.6	633
AB_0091	2.69	2621.3	974
AB_0097	2.37	1787.5	754
AB_0104	2.28	1841.2	808
AB_0111	2.56	1732.4	677
AB_0113	2.19	2461.0	1,124
AB_0116	2.01	1759.6	875
AB_0122	2.81	1777.9	633
AB_0173	2.50	1915.4	766
ABD_0028	2.14	3979.4	1,860
ABD_0034	1.59	2191.9	1,379
ABD_0059	1.64	1166.9	712
ABD_0085	1.74	2594.9	1,491
ABD_0162	2.11	2350.0	1,114
ABD_0164	1.61	1206.6	749
ABD_0178	2.05	2054.4	1,002
ABD_0181	2.14	1988.2	929
ABD_0235	1.66	1923.5	1,159
ABD_0240	2.05	2597.1	1,267
ABD_0260	1.99	1409.6	708
ABD_0261	2.34	2122.1	907
ABD_0283	1.50	1098.5	732
ABD_0295	2.14	1880.9	879
ABD_0301	1.74	1969.6	1,132
ABD_0335	1.70	1790.4	1,053

Supplementary Table 7: Copy number per leaf cell of chloroplast of all accessions in this study (Continued table)

SampleID	Whole genome sequence depth	Chloroplast genome sequence depth	Chloroplast copy number per cell
ABD_0338	1.58	1278.7	809
ABD_0341	2.14	1974.3	923
ABD_0356	2.00	1977.9	989
ABD_0357	1.40	1800.7	1,286
ABD_0435	1.64	1616.2	985
ABD_0451	1.45	1511.0	1,042
ABD_0467	1.59	2071.3	1,303
ABD_0477	1.59	2372.1	1,492
ABD_0506	1.70	1952.2	1,148
ABD_0535	1.92	2363.2	1,231
ABD_0544	1.64	2733.8	1,667
CS	2.30	2829.4	1,230
D0014	2.30	533.8	232
D0015	2.13	458.1	215
D0022	1.93	748.5	388
D0032	2.43	611.0	251
D0036	2.60	830.1	319
D0046	2.63	469.9	179
D0047	2.15	442.6	206
D0048	3.88	727.9	188
D0044	7.40	2431.6	329

Supplementary Table 8: CV error of each K values

K	1	2	3	4	5	6	7
CV error	0.89170	0.04811	0.06056	0.10783	0.13705	0.05544	0.09497

Supplementary Table 9: Number of pairwise measures of R^2

LSC	IRa	SSC	IRb
n=28	n=28	n=28	n=28
861 (0)	1431 (0)	NA	2556 (0)
0.2	0.11	NA	0.22
SD 0.36	SD 0.30	NA	SD 0.4

Note: Number of pairwise measures of R^2 and significant pairwise measures using the Fisher's exact test in brackets, mean R^2, and standard deviation.
